# Single‐Cell RNA‐Sequencing Provides Insight into Skeletal Muscle Evolution during the Selection of Muscle Characteristics

**DOI:** 10.1002/advs.202305080

**Published:** 2023-10-23

**Authors:** Doudou Xu, Boyang Wan, Kai Qiu, Yubo Wang, Xin Zhang, Ning Jiao, Enfa Yan, Jiangwei Wu, Run Yu, Shuai Gao, Min Du, Chousheng Liu, Mingzhou Li, Guoping Fan, Jingdong Yin

**Affiliations:** ^1^ State Key Laboratory of Animal Nutrition and feeding College of Animal Science and Technology China Agricultural University Beijing 100193 China; ^2^ Molecular Design Breeding Frontier Science Center of the Ministry of Education Beijing China; ^3^ Key Laboratory of Animal Genetics Breeding and Reproduction of Shaanxi Province College of Animal Science and Technology Northwest A&F University Yangling 712100 China; ^4^ Beijing National Day School Beijing 100039 China; ^5^ Key Laboratory of Animal Genetics College of Animal Science and Technology China Agricultural University Beijing 100193 China; ^6^ Nutrigenomics and Growth Biology Laboratory Department of Animal Sciences and School of Molecular Bioscience Washington State University Pullman WA 99164 USA; ^7^ National Animal Husbandry Service Beijing 100125 China; ^8^ Institute of Animal Genetics and Breeding College of Animal Science and Technology Sichuan Agricultural University Chengdu 625014 China; ^9^ Department of Human Genetics David Geffen School of Medicine University of California Los Angeles Los Angeles CA 90095 USA

**Keywords:** fibro‐adipogenic progenitors, muscle characteristics, neonatal myogenesis, pigs, single‐cell RNA‐sequencing

## Abstract

Skeletal muscle comprises a large, heterogeneous assortment of cell populations that interact to maintain muscle homeostasis, but little is known about the mechanism that controls myogenic development in response to artificial selection. Different pig (*Sus scrofa*) breeds exhibit distinct muscle phenotypes resulting from domestication and selective breeding. Using unbiased single‐cell transcriptomic sequencing analysis (scRNA‐seq), the impact of artificial selection on cell profiles is investigated in neonatal skeletal muscle of pigs. This work provides panoramic muscle‐resident cell profiles and identifies novel and breed‐specific cells, mapping them on pseudotime trajectories. Artificial selection has elicited significant changes in muscle‐resident cell profiles, while conserving signs of generational environmental challenges. These results suggest that fibro‐adipogenic progenitors serve as a cellular interaction hub and that specific transcription factors identified here may serve as candidate target regulons for the pursuit of a specific muscle phenotype. Furthermore, a cross‐species comparison of humans, mice, and pigs illustrates the conservation and divergence of mammalian muscle ontology. The findings of this study reveal shifts in cellular heterogeneity, novel cell subpopulations, and their interactions that may greatly facilitate the understanding of the mechanism underlying divergent muscle phenotypes arising from artificial selection.

## Graphical abstract

To date, little is known about the mechanism that controls muscle phenotype modification in response to artificial selection. Using single‐cell RNA sequencing, authors delineate panoramic muscle‐resident cell profiles with identification of novel cell subpopulations and cellular interactions in pigs (*Sus scrofa*) representing divergent muscle phenotypes. The findings should greatly facilitate the understanding of the mechanism of divergent muscle characteristics formation.

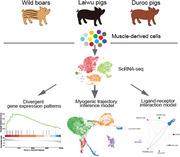

## 1 Introduction

The domestication of pigs (*Sus scrofa*) beginning 7000 years ago, combined with subsequent selective breeding, has resulted in huge phenotypic differences among breeds, especially in body shape and composition.^[^ ^1^ ^]^ Chinese pigs have greatly contributed to global pig breeding. Originally, Chinese pigs (obese‐type) were introduced into European countries in the 18th Century to improve pig fatness, driven by demands for energy‐rich food and tallow for candle manufacturing.^[^ ^2^ ^]^ Chinese pigs were re‐introduced in the 1980′s to improve pig reproductive performance.^[^ ^3^ ^]^ During the past 60 years, domestic pigs have been subjected to intense selection for lean meat ratio and growth efficiency, driven by increasing demands for animal protein in parallel with the desire for reduced caloric intake in modern societies. Consequently, the phenotypes of skeletal muscle have undergone significant changes in terms of muscle growth and mass, myofiber‐type composition, and intramuscular fat (IMF) accumulation.

Skeletal muscle, which accounts for ≈40% of body weight, plays vital roles in mechanics and metabolism throughout the lifetime of mammals, and resident cells are important for maintaining proper functioning. During early embryonic development, the paraxial mesoderm develops into myotomes, myogenic progenitors, and myoblasts, which then proliferate and fuse to form multinuclear‐myotubes. Fetal muscle development involves three distinct processes: myogenesis, adipogenesis, and fibrogenesis. The dynamics of these processes determine the cell composition of neonatal skeletal muscle and subsequent adult muscle phenotype.

All muscle fibers are formed during the fetal stage in pigs, and thus large numbers of stem/progenitor cells reside in neonatal muscle. Neonatal muscles represent a transitional stage bridging the resident cell profile of fetal muscle and adult muscle phenotypes.^[^ ^4^ ^]^

Genetic selection has led to large differences in muscle phenotypes among pig breeds. In wild boars, the original physiological characteristics of skeletal muscle have been preserved, endowing them with powerful strength, speed, stamina, and agility to survival in the wild. In contrast, Laiwu pigs, a domestic breed indigenous to China, have been selected for favorable meat quality accompanied by high fatness, with muscles characterized by relatively thin myofibers and a high level of IMF accumulation (≈10.3 ± 0.19%).^[^ ^5^ ^]^ Alternatively, Duroc pigs, one of the most popular lean‐type breeds, feature potent skeletal muscle growth potential and high lean meat yield, but relatively low IMF content (3.04 ± 0.33%).^[^ ^6^ ^]^ The mechanism underlying the development of these divergent muscle phenotypes is unclear.

The longissimus dorsi muscle is a representative muscle tissue for lean meat production assessment and meat quality evaluation in pig breeding. A large amount of data and knowledge based on this muscle tissue have been established, including histologic, genetic, transcriptomic, and epigenetic data concerning myogenic commitment, differentiation, and subsequent maturation.^[^ ^1^, ^6^, ^7^ ^]^

In this study, we selected the longissimus dorsi muscle to explore the shift in skeletal muscle‐resident cell profile underlying distinct muscle phenotypes resulting from domestication and artificial selection by employing scRNA‐seq analysis (scRNA‐seq). We anticipate that our findings will contribute to the current body of knowledge on the causality between muscle‐resident cell profiles and muscle phenotypes. This work also sheds light on the use of modulation of muscle‐resident cell profiles, transcriptomic expression, and ligand‐receptor interaction networks across cell types to improve muscle growth, muscle regeneration, and to develop therapeutic approaches for congenital myopathies.

## 2 Results

### 2.1 Major Cell Populations of Neonatal Skeletal Muscle

**Figure 1:**
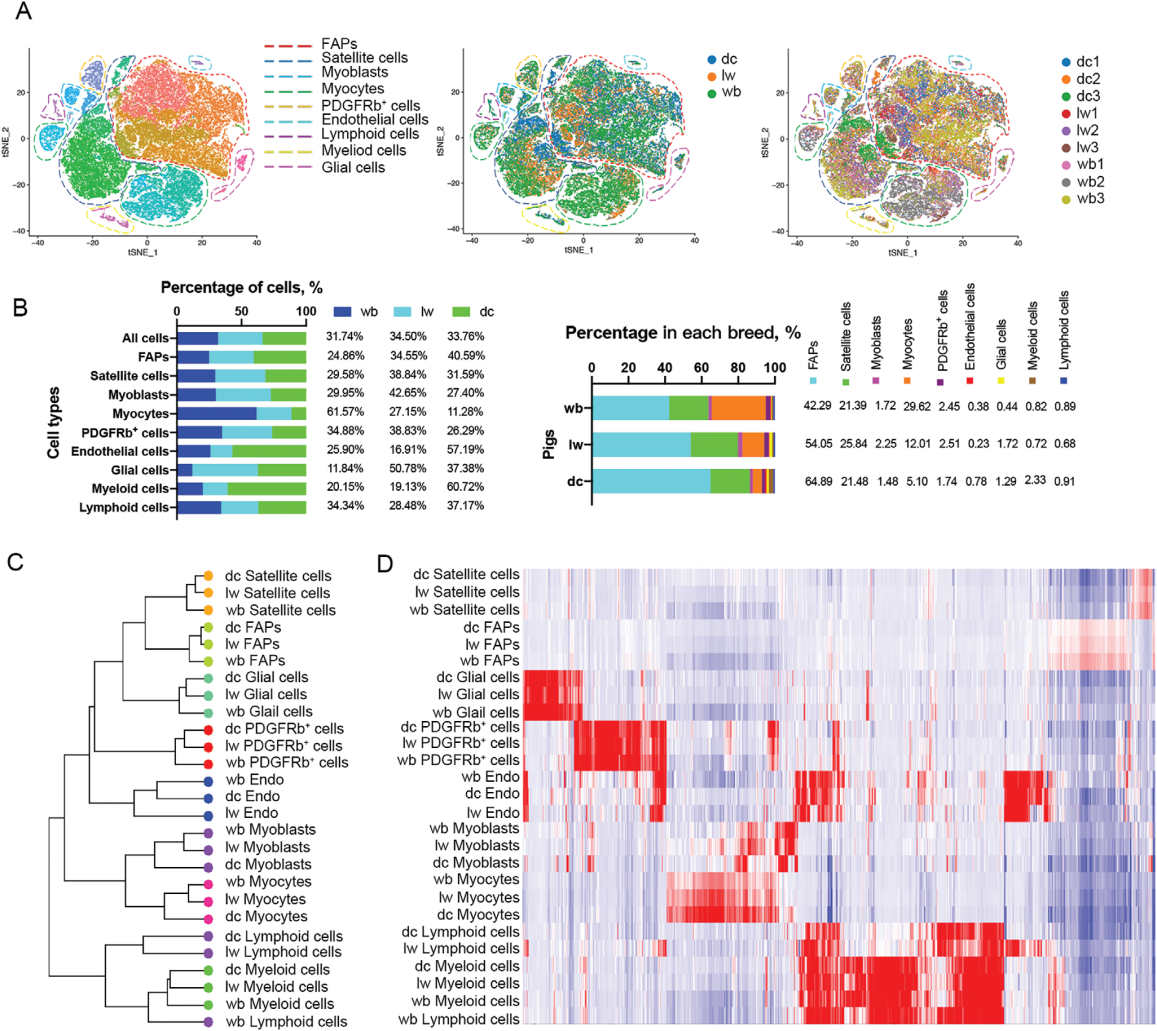
Single‐cell transcriptional profiling of wild boars, Laiwu pigs, and Duroc pigs skeletal muscle cells. A) t‐distributed stochastic neighbor embedding (t‐SNE) visualization of 60040 cells grouped by expression similarity and colored by cluster, breed, and donor. B) Distribution of skeletal muscle cell types in each breed (left) and inter‐breed heterogeneity revealed by plotting per‐breed major cell type distribution (right). C) Hierarchical clustering of major cell populations groups the cells by cell type rather than by organism. Cell‐type labels are as in Figure 1A; cell types were clustered using gene expression correlation. D) Heatmap showing gene orthologs similarly enriched within wild boars, Laiwu pigs, and Duroc pigs skeletal muscle cell types. dc: Duroc pigs, lw: Laiwu pigs, wb: wild boars.

Neonatal skeletal muscle cell populations from pigs were profiled using 10× Genomics scRNA‐seq. After the removal of cells with transcriptomes of subpar sample quality, a total of 60040 cells were captured for further analysis: 19054 cells from wild boars, 20716 cells from Laiwu pigs, and 20270 cells from Duroc pigs (Figure S1, Supporting Information). Information on quality control of the data, including total sequencing reads, UMI counts per cell, and total genes detected, is listed in Table S1 (Supporting Information). Using unsupervised clustering analysis, we identified nine cell populations across the breeds: fibro‐adipogenic progenitors (FAPs) (*DCN*, *COL1A1)*, but among them *PPARγ* was expressed at a low level; three myogenic lineages, including satellite cells (*PAX7*), myoblasts (*MYOG*), and myocytes (*ACTC1* and *TPM1*); PDGFRb^+^ cells (*PDGFRb* and *RGS5*); endothelial cells (*PECAM1* and *CDH5*); glial cells (*PLP1* and *MBP*); myeloid cells (*CSF1R* and *S100A8*); and lymphoid cells (*PTPRC* and *CD3G*) (**Figure** **1**A and Figure S1, Supporting Information). A relatively uniform distribution of the nine datasets from the three pig breeds in certain clusters indicated minimal batch effects.

Despite the evolutionary conservation of the composition of cell populations, we found remarkable divergence in the proportions of cell types among the breeds. Relative to wild boars, the proportion of FAPs in neonatal skeletal muscle of domestic pigs was increased while that of myocytes was decreased. Additionally, Duroc pigs had the highest proportions of endothelial cells and myeloid cells among the breeds by almost two‐fold, whereas Laiwu pigs had the highest proportion of glial cells (Figure 1B). Notably, the percentages of cell types in scRNA‐seq analysis were not directly equivalent to those detected by immunocytochemistry. For example, the proportion of satellite cells was 20%–26% in scRNA‐seq analysis (Figure 1B) and 6%–11% in immunocytochemical analysis (Figure S5B, Supporting Information). The discrepancy could be attributable to the cell dissociation procedure employed in scRNA‐seq, but also to different total numbers of cells between the two systems of analysis.

We quantified the transcriptome similarity of each homologous cell population and reconstructed a gene expression tree (Figure 1C,D). First, except for lymphoid cells, homologous cell populations shared high similarities in gene expression profiles across the pig breeds. Regarding wild boars, lymphoid cells first clustered with myeloid cells, then with lymphoid cells from domestic (Laiwu and Duroc) pigs, reflecting evolution of lymphoid cells and the similarity between lymphoid and myeloid cells. Except for myoblasts, myocytes, and endothelial cells, the similarities of homologous cell populations were higher between the two domestic breeds than between wild boars and either domestic breeds. These data suggest that skeletal muscle‐resident cell populations underwent more changes during domestication than during selective breeding. Notably, with respect to the similarity in gene expression of homologous myoblasts or myocytes, wild boars were closer to Laiwu pigs than to Duroc pigs. It might be attributable to the selection for lean growth efficiency, which broadened the gap of myogenic potential between lean‐type pigs and obese‐type pigs or wild boars. In summary, using a scRNA‐seq pipeline, we were able to identify dynamic resident cell populations underlying distinct muscle phenotypes.

### 2.2 Unbiased Dissection of FAP Subpopulations and Differences among Breeds

**Figure 2:**
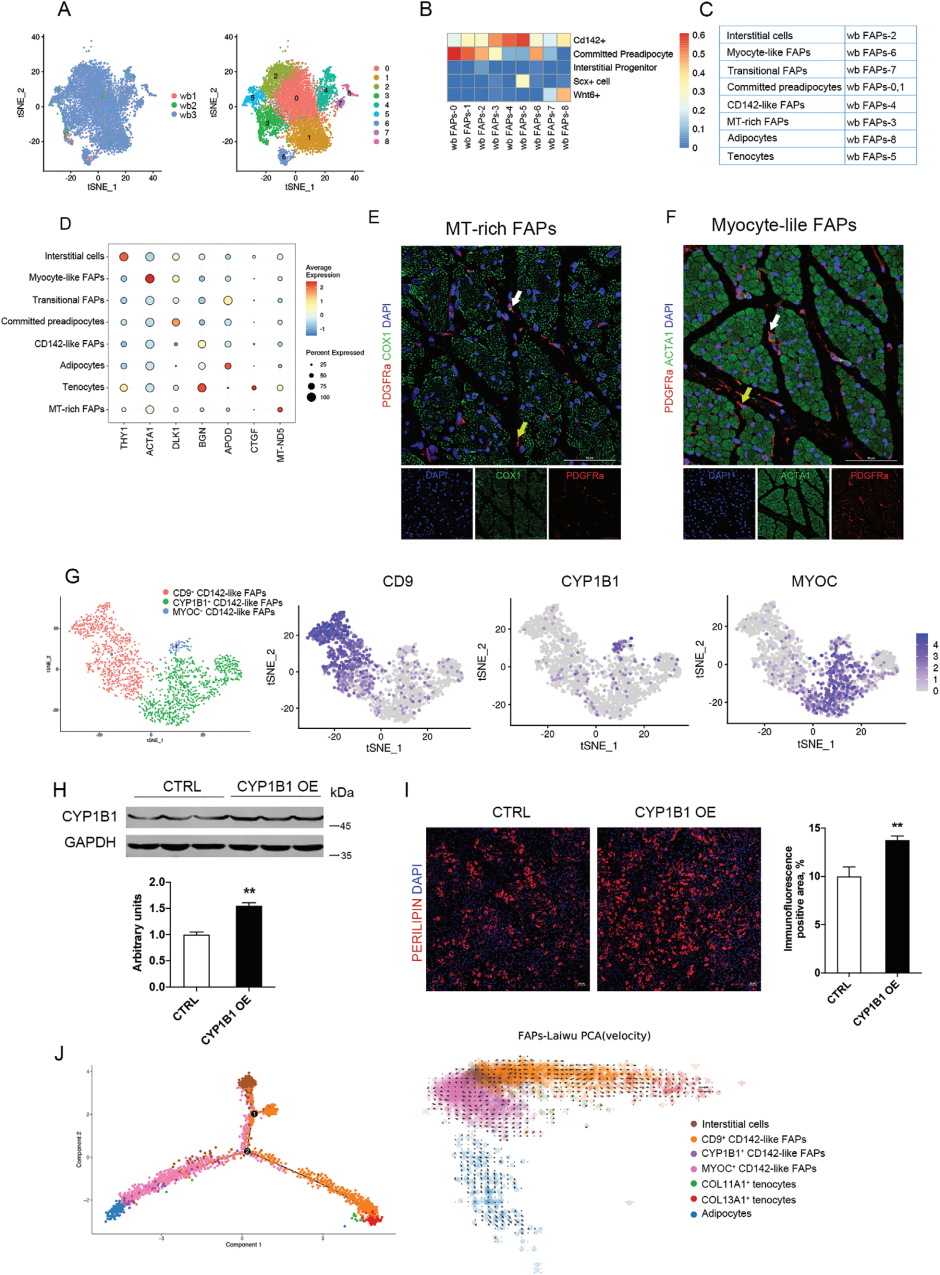
Characterization of the FAPs identified novel subpopulations. A) FAPs from wild boar skeletal muscle were selected and re‐analyzed. tSNE plot colored by donor (left) and serut subset (right). B) Comparison of observed wild boar muscle FAPs subsets to the FAPs previously reported.^[^ ^9^ ^]^ C) Summary of the annotation of FAPs subpopulations. D) Violin plots showing the expression levels and distribution of representative marker genes. E‐F) Representative confocal images of skeletal muscle stained for FAP marker PDGFRa (red), DAPI (blue), and MT‐rich FAPs marker COX1 (Green) in (E) and Myocyte‐like FAPs marker ACTA1 (green) in (F). White arrowheads denote FAPs that are positive for the marker (scale, 50 µm). Yellow arrowheads mark FAPs that are negative for expression of the marker. G) CD142‐like FAPs from Laiwu pigs were selected and re‐analyzed. t‐SNE plot colored by CD142‐like FAPs subsets (left) and showing the expression levels of markers for CD9^+^ CD142‐like FAPs (*CD9*), CYP1B1^+^ CD142‐like FAPs (*CYP1B1*) and MYOC^+^ CD142‐like FAPs (*MYOC*). H) The immunoblots presented here show the protein level of CYP1B1 in adipogenic precursors transfected with CYP1B1‐plasmid or empty vector 24 h in growth medium (left). Relative expression level was calculated (right). β‐tubulin was used as the internal control. Data were presented as means ± SEM (*n* = 3). The statistical significance of the difference between two means was calculated using *t*‐test, **P < 0.01. I) Immunofluorescent microscopy analysis of perilipin 1 in adipogenic precursors transfected with CYP1B1‐plasmid or empty vector at 7 d of adipogenic differentiation (left). Scale bars, 50 µm. Immunofluorescence (perilipin 1) positive area was calculated in right. Data were presented as means ± SEM (*n* = 3). The statistical significance of difference between the two means was calculated using *t*test, **P < 0.01. J) Pseudotime trajectories developed through Monocle analysis for CD142‐like FAP subpopulation (left). Developmental trajectory of CD142‐like FAPs subpopulations inferred by RNA velocity and visualized on the PCA projection (right).

FAPs account for the largest proportion of neonatal porcine muscle cells (Figure 1B) and play a vital role in muscle development,^[^ ^8^ ^]^ and therefore we further characterized subpopulations of FAPs across pig breeds using unsupervised clustering (**Figure** **2**A and Figure S2A,B, Supporting Information). Comparing neonatal muscle FAPs with those previously identified in both muscle and adipose tissues by similarity analysis based on overall gene expression profile combined with cell markers (Figure 2B‐D and Figure S2C,D, Supporting Information), we identified eight FAP subpopulations using existing annotations.^[^ ^9^ ^]^ In addition to the known subpopulations of FAPs, interstitial cells, committed preadipocytes, CD142‐like FAPs, transitional cells, adipocytes, and tenocytes, we identified two novel subpopulations: mitochondria (MT)‐rich FAPs and myocyte‐like FAPs. The markers for each FAP subpopulation are shown in Figure 2D. Notably, *PPARγ* was expressed at very low levels in adipocytes and at slightly higher levels in committed preadipocytes, suggesting that PPARγ is not an appropriate marker for these two cell types in neonatal skeletal muscle of pigs.

MT‐rich FAPs exhibited high expression of mitochondrial genes (*ND5*, *CYTB*, *COX2*) (Figure 2D,E), suggesting that this subpopulation is rich in mitochondria and characterized by active energy metabolism. Furthermore, the abundant expression of mitochondrial genes was a property not belonging to any of the other identified FAPs. Moreover, co‐immunostaining of MT‐specific proteins COX1 and TOMM20, and the FAP marker PDGFRα, confirmed the existence of higher numbers of mitochondria in the MT‐rich FAP subpopulation (Figure S2E, Supporting Information). Although further verification is required, it is reasonable to deduce that MT‐rich FAPs exist in neonatal skeletal muscle.

Myocyte‐like FAPs expressed both FAP markers (*DCN*, *COL1A1*) and myocyte markers (*ACTA1*, *TPM1*) (Figure 2D,F, and Figure S1, Supporting Information), which is different from both myofibroblasts^[^ ^10^ ^]^ and fibromyocytes.^[^ ^11^ ^]^ Myofibroblasts, which are specialized contractile fibroblasts, play an important role in connective tissue regeneration by producing extracellular matrix proteins.^[^ ^12^ ^]^ Relative to myofibroblasts, fibromyocytes have an opposite phenotypic trajectory, and exhibit a continuum in gene expression that ranges from profiles of contractile smooth muscle cells to those of fibroblast‐like cells.^[^ ^11^ ^]^ Notably, myocyte‐like FAPs expressed very low levels of ACTA2, a canonical marker of vascular smooth muscle cells,^[^ ^13^ ^]^ whereas myofibroblasts and fibromyocytes exhibit high expression of ACTA2. Additionally, myocyte‐like FAPs are not bipotent progenitors that were identified in a previous study,^[^ ^14^ ^]^ because myocyte‐like FAPs did not express the bipotent progenitors markers *pax3* and *myf5*. Furthermore, bipotent progenitor cells can be further clustered into two cell subsets that express myogenic markers or connective tissue markers.

The CD142^+^ FAP subpopulation (Figure S2F, Supporting Information) shares markers of committed preadipocytes (*DLK1*, *LPL*, *VCAM1*) and resembles the gene expression profile of committed preadipocytes (Figure 2B and Figure S2C,D, Supporting Information), but differs from the CD142^+^ cells from mice.^[^ ^9a^ ^]^ Thus, we classified the CD142^+^ subpopulation into committed preadipocytes. We also identified a specific FAP subpopulation named as CD142‐like FAPs, whose overall gene expression profile resembles CD142^+^ cells but does not express CD142 gene.

The subpopulation of CD142‐like FAPs is heterogeneous and can be characterized into three subclusters: CYP1B1^+^ CD142‐like FAPs, CD9^+^ CD142‐like FAPs, and MYOC^+^ CD142‐like FAPs (Figure 2G). CYP1B1 plays an important role in adipogenesis and obesity.^[^ ^15^ ^]^ Thus, CYP1B1^+^ CD142‐like FAPs probably possess adipogenic potential. We demonstrated that overexpression of CYP1B1 in porcine primary FAPs promoted adipogenic differentiation (Figure 2H, I), suggesting that CYP1B1^+^ CD142‐like FAPs contributed to high IMF content. Furthermore, the absence of this subpopulation might be implicated in low IMF content.

CD9^high^/PDGFRA^+^ progenitors exhibit fibrotic potential,^[^ ^16^ ^]^ indicating that CD9^+^ CD142‐like FAPs might also possess fibrogenic potential. Trajectory analysis revealed the heterogeneity of CD142‐like FAPs and their subsequent differentiation pathways (Figure 2J). Reportedly, CD142 cells play paradoxical roles in adipogenesis,^[^ ^9a,b^ ^]^ which might be due to the heterogeneity of CD142 FAPs as shown in this study.

**Figure 3:**
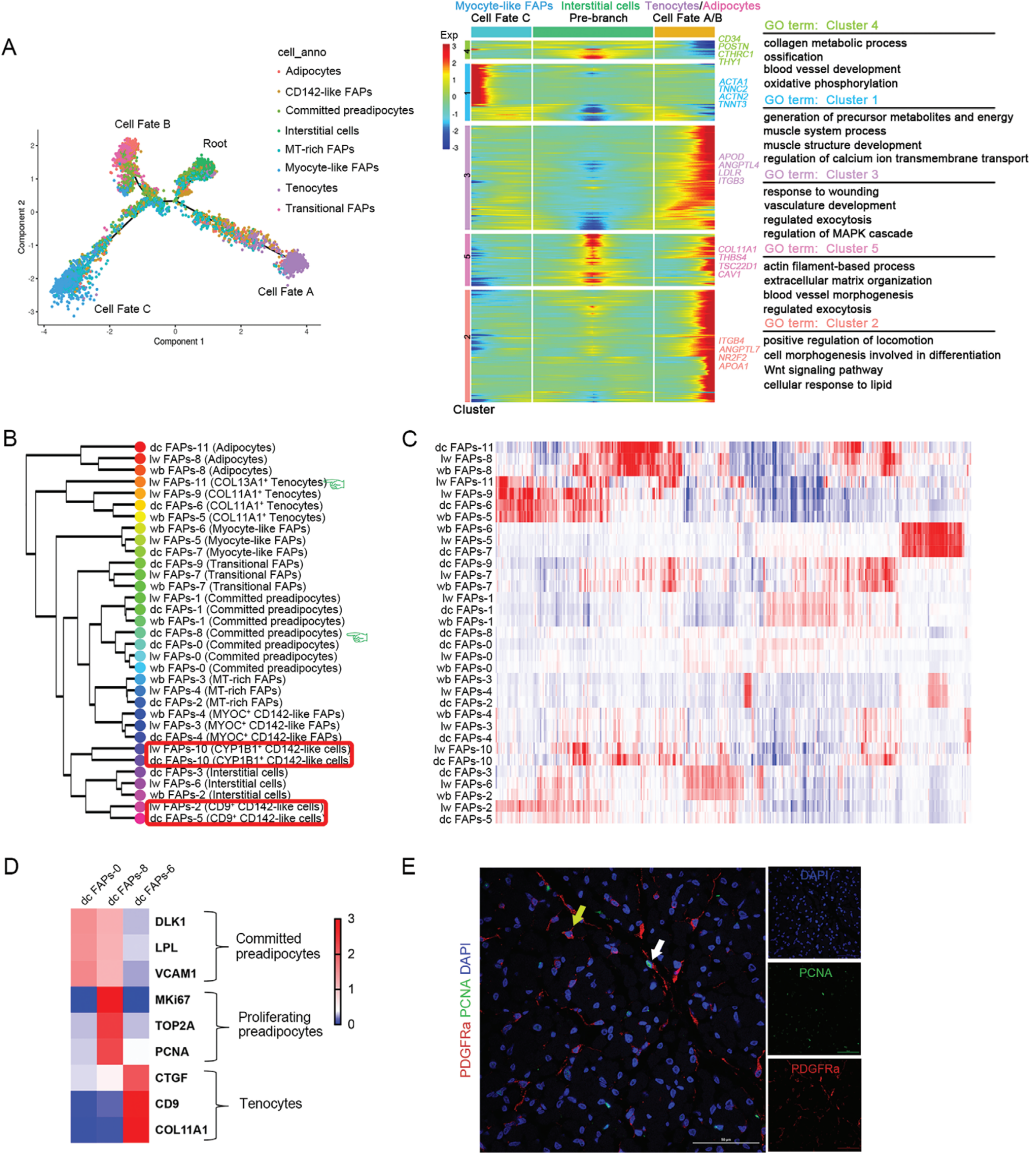
Pseudotime analyses of FAPs and the heterogeneity of FAP subpopulations among pig breeds. A) Pseudotime ordering of all FAPs subpopulations. Each dot represents one cell and each branch represents one cell state (left). Heatmap illustrating the differentially expressed genes (DEGs) dynamics towards myocyte‐like FAPs and tenocytes/adipocytes fate along pseudotime (right). The DEGs were clustered into 5 gene sets according to k‐means. GO terms enriched for each gene set were labeled in the right panel. Tenocytes FAPs fated represents cell fate A, adipocytes fated represents cell fate B, while myocyte‐like FAPs represent cell fate C. B‐C) Comparison of wild boars, Laiwu pigs, and Duroc pigs FAPs subsets. (B) Orthologous wild boar, Laiwu pig, and Duroc pig subpopulations were established by hierarchal clustering. (C) Heatmap showing genes similarly enriched within wild boars, Laiwu pigs, and Duroc pigs. D) Heatmap of differentially expressed genes across dc FAPs‐0 and 8 subsets (both defined as committed preadipocyte) and a dc FAPs‐6 (defined as tenocyte) as a control. E) Immunofluorescent images of PDGFRa^+^PCNA^+^ FAPs in pig skeletal muscle. White arrowheads denote FAPs that are positive for PCNA (scale, 50 µm). Yellow arrowheads mark FAPs that are negative for expression of PCNA. dc: Duroc pigs, lw: Laiwu pigs, wb: wild boars.

Pseudotemporal analysis of FAPs suggested that interstitial cells may be fated to fibrogenic differentiation (cell fate A), adipogenic differentiation (cell fate B), or myogenic differentiation (cell fate C) (**Figure** **3**A, left). To examine the sequential gene expression profile along each branch, we visualized gene expression dynamics along the pseudotime trajectory of FAPs, identifying five distinct gene sets based on expression patterns (Figure 3A, right).

Next, we investigated key transcription factors (TFs) involved in FAP fate decision‐making (Figure S2G, Supporting Information). TFs such as *GLI1*, *IRF3*, and *HES1* were enriched in the tenocyte branch, while *STAT1* and *ID2* were identified in the adipocyte branch. These TFs are known to be important for fibrogenesis and adipogenesis, respectively. Their involvement in the activation of signaling pathways is essential for fibrogenesis (Hedgehog, Toll‐like receptor, and Notch pathways) and adipogenesis (JAK‐STAT1, TGFβ). Additionally, these TFs show breed‐specific expression, indicating that they may be key regulators of FAP commitment and the divergent capacities of IMF accumulation among pig breeds.

Most homologous FAP subpopulations are conservative, showing a one‐to‐one relationship among breeds in hierarchical clustering analysis (Figure 3B, C). However, there were several breed‐specific FAP subpopulations in Laiwu and Duroc pigs, indicating the heterogeneity of FAP subpopulations among pig breeds. In neonatal domestic pigs, FAPs are more heterogeneous than in wild boars. We identified the proliferating preadipocytes (dc FAPs 8) specifically found in the skeletal muscle of neonatal Duroc pigs (Figure 3B). In addition to the canonical preadipocyte markers *DLK1*, *VCAM1*, and *LPL*, proliferating preadipocytes showed high expression of cell proliferation markers *PCNA*, *TOP2A*, and *MKI67* (Figure 3D, E). Consistently, using immunofluorescence staining of neonatal muscle, far fewer proliferating preadipocytes were observed in Laiwu pigs and wild boars than in Duroc pigs (Figure S3A, Supporting Information). These data suggest that Duroc pigs have more proliferating preadipocytes than the other breeds, which might be attributable to the prolonged proliferation of preadipocytes in Duroc pigs.

Greater numbers of proliferating preadipocytes in neonatal skeletal muscle of Duroc pigs implied that these pigs retain some genetic advantage that allows them to achieve high IMF accumulation under certain conditions. Alternatively, the lower IMF content of Duroc pigs relative to that of Laiwu pigs is not necessarily inconsistent because the IMF content in skeletal muscle of adult pigs is mainly controlled by adipocyte hypertrophy rather than adipocyte hyperplasia.^[^ ^17^ ^]^ Subsequently, we explored the mechanisms underlying the differences in adipogenic differentiation among the three pig breeds in terms of cell interactions and gene expression patterns of muscle‐resident cells.

In wild boars, the CD142‐like FAPs contained only MYOC^+^ CD142‐like cells. However, in domestic pigs, the CD142‐like FAPs included two more subpopulations: CD9^+^ CD142‐like FAPs and CYP1B1^+^ CD142‐like FAPs (Figure 3B). The gene signatures of CD9^+^ CD142‐like FAPs and CYP1B1^+^ CD142‐like FAPs varied across the breeds (Figure 2G and Figure S3B, Supporting Information).

The expansion of adipose depots is driven by adipocyte hyperplasia and hypertrophy. Adipocyte hyperplasia mainly occurs at the late fetal to early postnatal stages, whereas adipocyte hypertrophy drives fat accumulation in adipose tissue in adults. In this study, FAP subpopulations varied significantly among the breeds (Figure S3C, Supporting Information). No significant differences in adipocyte numbers were found among the breeds, suggesting that adipocyte hyperplasia in the skeletal muscle during fetal development did not vary between domestic pigs and wild boars. Therefore, the divergence in IMF accumulation between obese‐type and lean‐type pigs may originate from distinct potentials of postnatal adipocyte hypertrophy and/or hyperplasia rather than fetal‐stage adipocyte hyperplasia. Additionally, a lack of CYP1B1^+^ CD142‐like FAPs might be responsible for the low IMF content in adult wild boars.

Regarding tenocytes, the clustering distance between wild boars and Duroc pigs was the closest among the breeds (Figure 3B). We identified a novel subpopulation of breed‐specific COL13A1^+^ tenocytes (lwFAPs‐12) in the neonatal skeletal muscle of Laiwu pigs (Figure S3D, Supporting Information). Collagen XIII is a postsynaptic/synaptic neuromuscular junction component.^[^ ^18^ ^]^ Endostatin (encoded by *COL18A1*) ameliorates fibrosis in the liver, lungs,^[^ ^19^ ^]^ and other organs.^[^ ^20^ ^]^ Meanwhile, COL13A1^+^COL18A1^+^ tenocytes exhibited high expression of genes enriched in GO biological process in terms of nervous system development and angiogenesis. These data suggested that COL13A1^+^COL18A1^+^ tenocytes participate in the formation of the neuromuscular junction and the amelioration of fibrosis.

Collectively, we profiled neonatal muscle‐resident FAPs to identify two novel subpopulations: MT‐rich and myocyte‐like FAPs. Subpopulations of FAPs in domestic pigs became more heterogeneous and breed‐specific relative to those in wild boars. Findings concerning the variation in FAP populations, the existing continuum between FAPs and myocytes, and the differential expression of TFs suggested that the capacities for IMF accumulation are mainly mediated by the dynamics of myogenesis, adipogenesis, and fibrogenesis.

### 2.3 Identification and Trajectory Analysis of Myogenic Lineages in Neonatal Pigs

**Figure 4:**
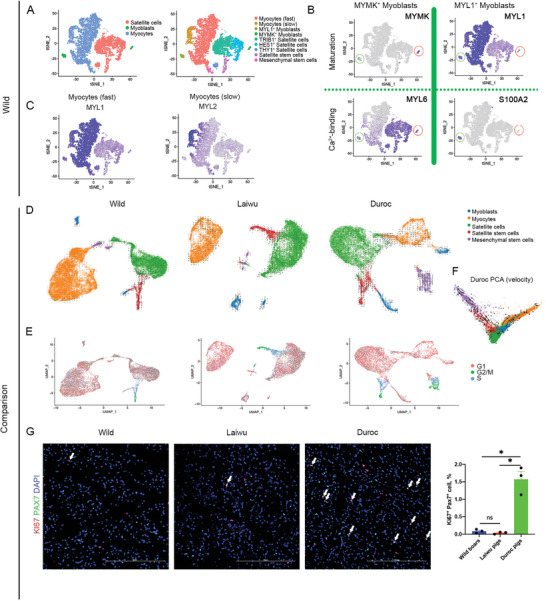
Identification and trajectory analysis of myogenic lineages in neonatal pigs. A) Myogenic lineage cluster (satellite cells, myoblasts, and myocytes) from wild boars were selected and re‐analyzed. tSNE plot colored by myogenic lineage cluster (left) and manual classified cell subsets (right). B) t‐SNE maps showing the expression levels of genes related to muscle maturation and Ca^2+^ binding capacity in two myoblast subpopulations. C) Fast and slow myocytes recognized by established myofiber type‐specific markers (*MYL1* and *MYL2* respectively). D, F) Developmental trajectory of myogenic lineages subpopulations inferred by RNA velocity and visualized on the UMAP (D) and PCA projection (F). E) UMAP map displaying cell cycle stage of each cell [S (blue), G2/M (green), G1 (pink)] assigned by the CellCycleScoring function in Seurat. G) Representative confocal images of PAX7 (green) and KI67 (red)‐ immunostained skeletal muscle of wild boars, Duroc, and Laiwu pigs (scale, 200 µm). Percentage of KI67^+^PAX7^+^ cells (normalized to DAPI cells) in wild boars (blue), Laiwu (red), and Duroc pigs (green). Data were presented as means ± SEM (*n* = 3), *P < 0.05.

Using unsupervised clustering of myogenic lineages (satellite cells, myoblasts, and myocytes) of each breed (**Figure** **4**A and Figure S4A,B, Supporting Information), we defined a hierarchy of myogenic lineages that included four subpopulations: committed mesenchymal stem cells (MSCs) marked by expression of *CD34*, *CD73*, and *CD90*; satellite cells marked by *PAX7*; myoblasts by *MYOG*; and myocytes by *ENO3* (Figure S4C, Supporting Information). Notably, during mouse embryonic/fetal ontogeny, PAX7^+^ myogenic stem cells have been shown to express MyoD1 and Mrf4 during the satellite cell (PAX7^+^) specification that begins during embryogenesis.^[^ ^21^ ^]^ However, in our pig neonatal muscle, expression levels of MYOD1 and MRF4 differed between quiescent satellite cells (HES1^+^ satellite cells, TRIB1^+^ satellite cells, and PAX7^+^ myogenic cells) and proliferating satellite cells (satellite stem cells and PAX7^+^ myogenic cells). Quiescent satellite cells showed higher expression of MRF4 and lower expression of MYOD1 compared with proliferating satellite cells.

Satellite cells, myoblasts, and myocytes exhibited heterogeneity. For example, satellite cells were grouped into four subpopulations (Figure 4A and Figure S4A,B,D, Supporting Information). The two major subpopulations included TRIB1^+^ satellite cells, which distinguished by high expression of genes promoting skeletal muscle cell differentiation (GO: 00 35914, *P* = 3.9 × 10^−4^), and HES1^+^ satellite cells, which were distinguished by expression of genes that suppress myogenic differentiation (e.g., *HES1*, *DDIT4*, and *TSC22D3*). Notably, compared with other myogenic lineages, satellite stem cells exhibited strong expression (> 100‐fold) of cell cycle genes (*MKI67*, *CCNB2*, and *PCNA*). THY1^+^ satellite cells expressed markers of satellite cells (*PAX7*) and MSCs (*THY1* and *CFD*), representing the transitional state from MSCs to satellite cells, and were specific to wild boars.

We also identified MYMK^+^ and MYL1^+^ myoblasts, two subpopulations that were considered to represent the early and late stages of differentiation, respectively (Figure 4B and Figure S4E,F, Supporting Information), based on known MYMK expression in confluent myoblasts^[^ ^22^ ^]^ and MYL1 expression in myocytes. MYMK^+^ and MYL1^+^ myoblasts possess distinct Ca^2+^‐binding capacity. MYL1^+^ myoblasts show high expression of S100A2, which contains two EF‐hand calcium‐binding motifs, whereas MYMK^+^ myoblasts show high expression of genes that encode proteins with weak ability to bind calcium ions (*MYL6*), which is consistent with the regulatory roles of calcium signaling in myoblast differentiation.^[^ ^23^ ^]^ Furthermore, myocytes included fast myocytes and slow myocytes with high expression of *MYL1* and *MYL2*, respectively, corresponding to fast‐twitch and slow‐twitch fibers (Figure 4C and Figure S4G,H, Supporting Information).

Although previous studies revealed the hierarchy of myogenic lineages during muscle regeneration in adult humans and mice,^[^ ^24^ ^]^ the hierarchy of neonatal myogenic lineages remains unclear.^[^ ^25^ ^]^ We performed pseudotime analysis and profiled the cell cycle state of the myogenic lineages and found differences in both the trajectories and cell cycling of myogenic lineages among the breeds (Figure 4D‐F). Most satellite cells are quiescent, while the neonatal pool of muscle satellite cells continues to expand, mainly relying on the myogenic differentiation of both MSCs and satellite stem cells.

Skeletal muscle MSCs were mainly retained in the S and G2/M phases in neonatal Duroc pigs, in the G1 phase in wild boars, and in the intermediate phase in Laiwu pigs (Figure 4E). The divergence in myogenic trajectories among the breeds illustrated that most of the MSCs in the two domestic breeds kept proliferating, whereas most of the MSCs in neonatal wild boars differentiated into satellite cells and myocytes (Figure 4D). This result also supports the possibility that MSCs are associated with myocytes, as shown in Duroc pigs (Figure 4A), warranting further study.

Remarkably, neonatal satellite stem cells possessed the potential to proliferate or asymmetrically divide into daughter quiescent satellite cells and committed progenitors (Figure 4D, E). RNA velocity with PCA embedding is shown in Figure 4F and UMAP embedding in Figure 4D. Although velocity with PCA embedding is a mathematically simpler representation, it can provide the transformation relationship between two cells that are not adjacent in the UMAP map. Interestingly, RNA velocity analysis with PCA embedding suggested that MSCs might preserve the capability to mutually transform with satellite stem cells, especially in neonatal Duroc pigs (Figure 4F), in addition to directly differentiating into satellite cells or myocytes. Notably, our analysis only provides the possible transformation relationship between cells, and thus requires further verification. The above results suggest that the myogenic lineages of wild boars possess low proliferation potential relative to those of the two domestic breeds, and were corroborated by immunofluorescent images (Figure 4G).

The asymmetric division of satellite stem cells can generate one committed myoblast and one quiescent satellite cell.^[^ ^26^ ^]^ In turn, the committed PAX7^–^MYOG^+^ myoblasts can give rise to PAX7^+^MYOG^–^ and PAX7^+^MYOG^+^ daughter cells.^[^ ^27^ ^]^ Interestingly, trajectory analysis suggested that the two opposite directions of myogenic differentiation might exist among the breeds; that is, satellite stem cells might generate myoblasts in wild boars and Laiwu pigs, while myoblasts might generate satellite stem cells in Duroc pigs (Figure 4D). We did not carry out a kinetics study to trace cell subpopulation transformation across the developmental stages. However, considering the vigorously developing neonatal skeletal muscle in which a variety of cells are present at one point in development, it makes sense to explore the trajectory of cell differentiation to depict relationships among the cells in this tissue.

**Figure 5:**
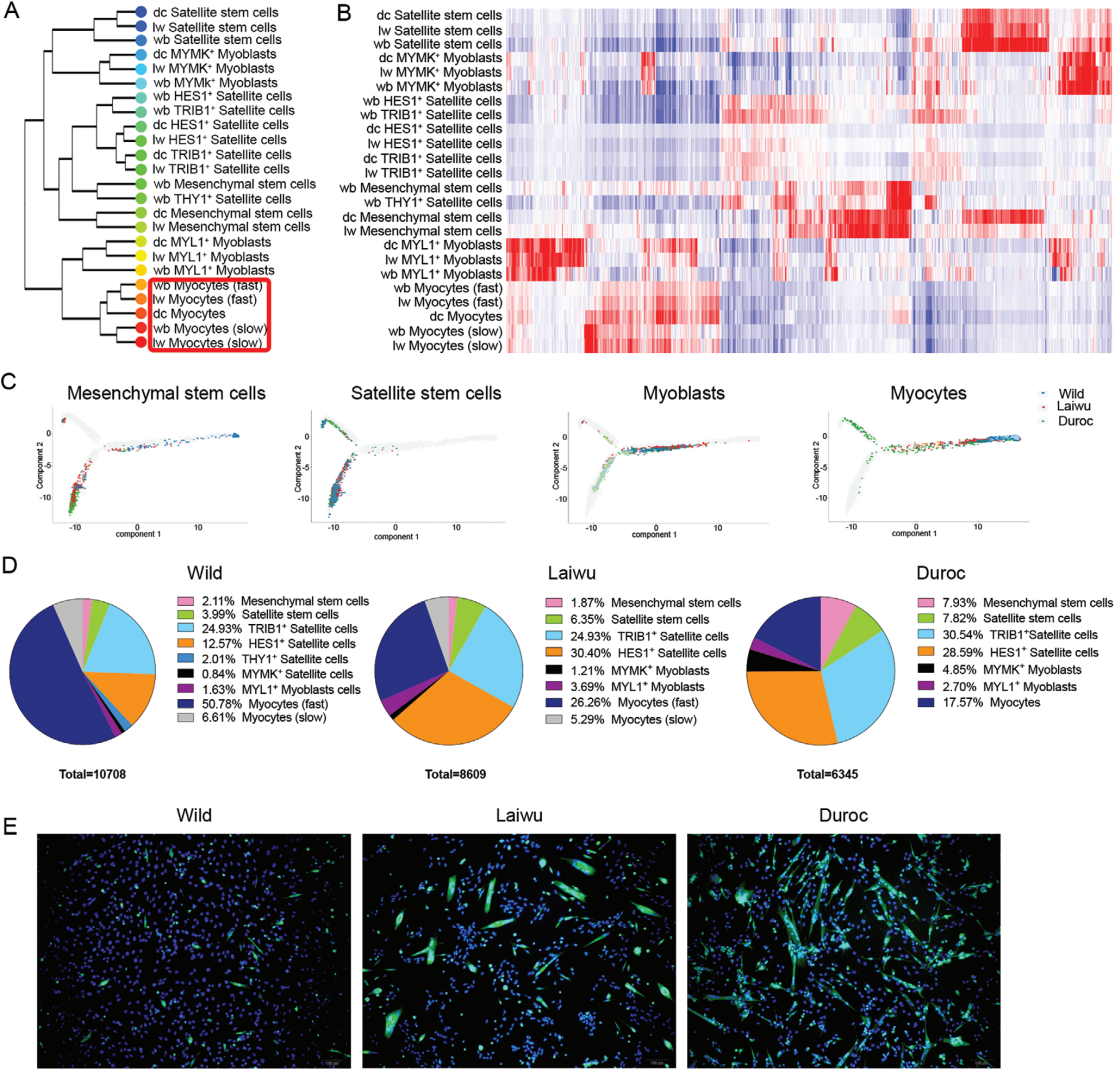
Comparison of heterogeneity and myogenesis potential of myogenic lineages among pig breeds. A‐B) Comparison of wild boars, Laiwu, and Duroc pig myogenic lineage subpopulations. C) Monocle reverse graph showing a joint trajectory comparing the myogenic trajectories of three pig breeds of indicated myogenic lineage subpopulations. Graph colored by pig breeds. D) Distribution of myogenic lineage subpopulations in each breed. E) Immunofluorescent microscopy analysis of the morphological changes and expression of MYOSIN in myogenic precursors of wild boars, Duroc, and Laiwu pigs at 48 h of differentiation. dc: Duroc pigs, lw: Laiwu pigs, wb: wild boars.

We thus constructed phylogenetic trees of the myogenic lineages by unsupervised hierarchical clustering (**Figure** **5**A, B). First, MSCs clustered with satellite cell subpopulations (excluding satellite stem cells), suggesting that MSCs may be able to generate a population of resting satellite cells, such as TRIB1^+^, HES1^+^, and THY1^+^ satellite cells. Second, satellite stem cells formed a branched clade with MYMK^+^ myoblasts, hinting that asymmetric division of satellite stem cells produces committed myoblasts. Third, the mature myogenic lineages, myocytes, and myoblasts (MYL1^+^) appeared as a clear outgroup distinct from all other subpopulations, indicating that satellite cells were derived from the two above pathways and eventually differentiated into myoblasts (MYL1^+^) and myocytes (slow and fast). Moreover, the phylogenetic trees reflected that the clustering distances of each myogenic subpopulation were closer between the domestic breeds than between either domestic breed and wild boars, with the exception of myocytes.

Relative to domestic pigs, neonatal satellite cells from wild boars appeared more heterogeneous (Figure 5A), as demonstrated by wild boar‐specific THY1^+^ satellite cells. Furthermore, myocytes in neonatal wild boars and Laiwu pigs were classified into fast and slow myocytes, while those in neonatal Duroc pigs displayed a continuum that could not be independently further classified, despite observing both fast and slow myofibers under immunofluorescence staining of muscle tissue sections (Figure S5A, Supporting Information).

A joint trajectory was built across the breeds (Figure 5C). Myogenic lineages of neonatal Duroc pigs were distributed more closely to the original point on the joint pseudotime trajectory than those of both neonatal wild boars and Laiwu pigs. Similarly, in the domestic pig breeds, the proportion of stem cells in the neonatal muscle was increased and the proportion of differentiated terminal cells, such as myoblasts and myocytes, was decreased accordingly (Figure 5D). Despite satellite stem cells among the three breeds being mainly in the S and G2/M phases, the fewest satellite stem cells were observed in wild boars (Figure 5D). These data suggested that domestic pigs possess a higher potential for myogenesis than wild boars, which was confirmed by immunofluorescent staining (Figure 5E). To exclude the influence of the number of satellite cells on their differentiation potential, we quantified the numbers of PAX7^+^ cells among the myogenic cells derived from each breed, and found no significant differences in the ratios of PAX7^+^ cells to total cells (Figure S5C, Supporting Information). Furthermore, we did not observe significant differences in the proliferation of myogenic precursor cells derived from neonatal skeletal muscle of the different breeds, although the proliferation of myogenic precursor cells from Duroc pigs tended to be higher than that of wild pigs (*P* = 0.09, Figure S5D, Supporting Information). Therefore, together with the percentage of myosin‐positive myotubes, our findings demonstrated that domestic pigs have a higher myogenic potential than wild boars.

These data illustrate the panorama of neonatal myogenic lineages, and may facilitate the deciphering of the differentiation trajectory of myogenic subpopulations. They also suggest that the pursuit of specific muscle characteristics led to reduced heterogeneity of satellite cells and asynchronous differentiation in domestic pig breeds relative to wild boars.

### 2.4 Immune Cells in the Skeletal Muscle

Muscle‐resident immune cells influence myogenesis, including satellite cell activation and differentiation, as well as muscle regeneration. In neonatal porcine skeletal muscles, immune cells account for ≈2%–3% of the total cell population (Figure 1B). Here, we clustered immune cells including myeloid cells and lymphoid cells into 11 subpopulations across the breeds: mature macrophages (MACs), proliferating macrophages (Pro‐MACs), CD4^+^ T cells, mature NK/T cells, proliferating NK/T (Pro‐NK/T), neutrophils 1 (Neu 1), neutrophils 2 (Neu 2), hematopoietic stem cells (HSCs), mast cells, monocytes, and B cells (Figure S6A, Supporting Information). The expression of marker genes defining the immune subpopulations are shown in Figure S6B (Supporting Information).

The variation in the proportions of immune cell subpopulations across the breeds reflected the impacts of pig domestication and selection on the immune system. Among the three breeds, Duroc pigs had the highest proportion of immune cells in skeletal muscle (Figure 1B), which might be a consequence of genetic selection for lean meat growth and modern intensive feeding systems in the pig industry.

There are two subsets of neutrophils in neonatal skeletal muscle: Neu 1, which comprises the majority of cells; and Neu 2, a rare subpopulation that exhibits high expression of type I interferon response genes, including *IFIT1*, *IRF7*, and *RSAD2* (Figure S6B, Supporting Information). The proportions of the two subpopulations varied significantly among the breeds (Figure S6C, Supporting Information). The proportion of Neu 1 showed a gradual decrease from 28.33% of the total immune cells in wild boars to 17.26% in Laiwu pigs and 5.53% in Duroc pigs. The proportion of Neu 2 was higher in Duroc pigs (5.68%) than in wild boars (0.57%). Notably, Neu 2 was not observed in Laiwu pigs. Because Neu 2 cells exhibit strong a type I interferon response, the variation in the proportions of Neu 2 in this study may reflect the different capacities of innate immunity among the pig breeds, which is consistent with a previous study.^[^ ^6^ ^]^ Similarly, the distribution of T cells varied among the breeds (Figure S6C, Supporting Information). Laiwu pigs had the largest number of CD4^+^ T cells and the fewest NK/T cells, with no differences between wild boars and Duroc pigs. Similar to our results, studies previously reported that, under obese conditions, the number of NKT cells was reduced^[^ ^28^ ^]^ whereas that of CD4^+^ T helper 1 cells was increased,^[^ ^29^ ^]^ suggesting that variation in the proportions of NK/T cells and CD4^+^ T cells might be correlated to fatness.

### 2.5 The Ligand‐Receptor Interaction Network across Skeletal Muscle‐Resident Cell Types

**Figure 6:**
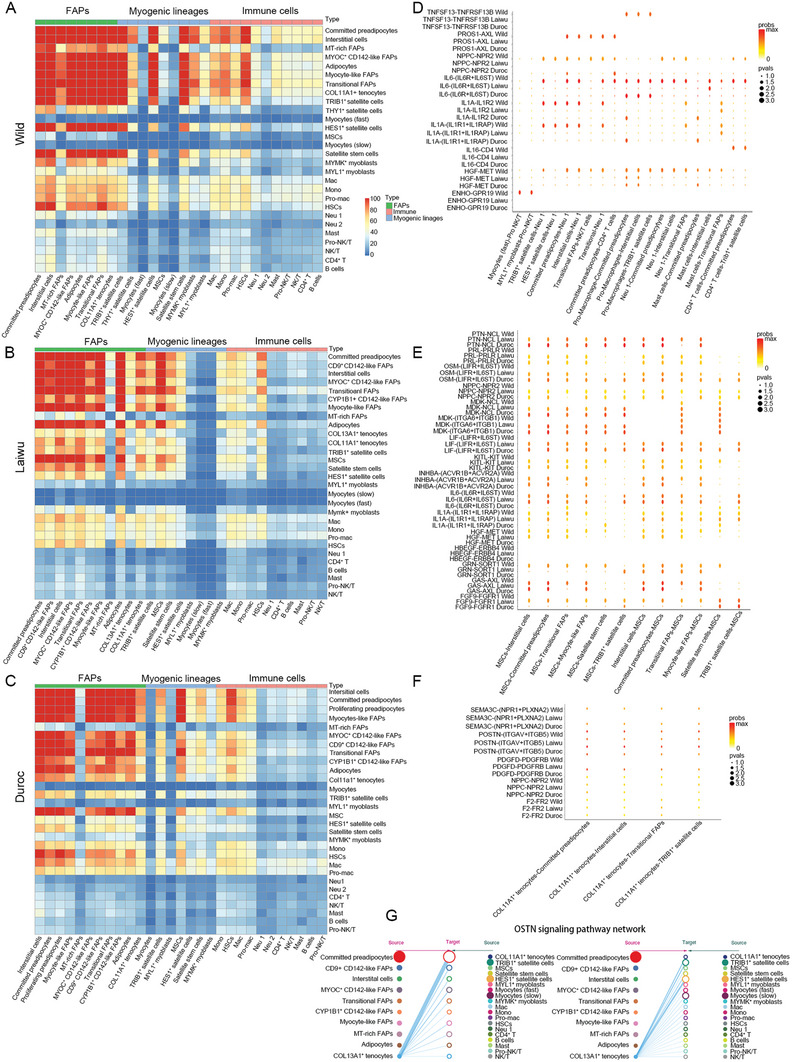
The ligand‐receptor interaction network analysis across skeletal cell types. A‐C) Heat map depicting the number of all possible interactions between the clusters analyzed in wild boars (A), Laiwu pigs (B), or Duroc pigs (C). Cell types are grouped by broad lineage (FAPs, myogenic lineages cells, or immune cells). D‐F) Dot plot depicting selected ligand‐receptor interactions between immune cell subpopulations and other cell types (D), mesenchymal stem cells(MSCs) and other cell types (E), COL11A1^+^ tenocytes, and other cell types (F). An interaction is indicated as color‐filled circle at the cross of interacting cell types in a tissue (x‐axis) and a ligand‐receptor pair (*y*‐axis), with circle size representing the significance of −log10P values in a permutation test and colors representing the means of the average expression level of the interacting pair. G) Hierarchical plot shows the inferred intercellular communication network (a case of specific OSTN signaling pathway in Laiwu pigs). Solid and open circles represent source and target, respectively. Circle sizes are proportional to the number of cells in each cell group. Edge colors are consistent with the signaling source.

Different compositions of cell types can result in different cellular communication networks. To explore the variation in ligand‐receptor interaction networks underlying the distinct muscle phenotypes, we performed a ligand‐receptor interaction analysis that led to the identification of 50 signaling pathway networks in the skeletal muscle (**Figure** **6** and Figure S7, Supporting Information).

FAPs, as an interaction hub, provided a source of the cytokines adrenomedullin (ADM), IFN gamma (IFNG), and granulin (GRN), which regulate activities of satellite cells, immune cells, and glial cells, respectively (Figure S7A,B, Supporting Information). Activation of calcitonin receptor (CALCR), the ADM receptor, is critical for maintaining quiescence in muscle stem cells,^[^ ^30^ ^]^ and IFNG functions to activate MACs.^[^ ^31^ ^]^ In addition, various growth factors, interleukins (ILs), TGFb superfamily, and TNF family members that are secreted from satellite cells, immune cells, or FAPs, regulate the proliferation (e.g., hepatocyte growth factor, HGF) and fibrogenic (e.g., myostatin) or adipogenic differentiation of FAPs (e.g., colony‐stimulating factors, CSF)^[^ ^32^ ^]^ (Figure S7C,D, Supporting Information).

We also conducted cell interaction analysis of FAP subpopulations, myogenic lineages, and immune cell subpopulations. The results showed that, in domestic pigs, the numbers of presumed interactions involving immune cell subpopulations and MT‐rich FAPs were significantly decreased, and those between MSCs and other cell types increased, relative to wild boars (Figure 6A‐E).

The ligand‐receptor interaction analysis of MSCs supported the hypothesis that the myogenic lineages of Duroc pigs were more proliferative than those of wild boars. Specifically, Duroc pigs exhibited higher expression of the growth factor (*MK*, *PTN*, *EGF*, *FGF*, *GAS*, *HGF*) and cytokine (*OSM*, *PRL*, *IL1*, *IL6*, *ACTIVIN*, *LIF*) genes responsible for cell proliferation^[^ ^33^ ^]^ than wild boars (Figure 6E).

Compared with domestic pigs, immune cell subpopulations in wild boars showed more interactions with other cell types via a wealth of cytokines, especially within pathways involving TNF superfamily memeber 13 (APRIL), energy homeostasis associated (ENHO), and neuropeptide Y (NPY), which were specifically present in wild boars (Figure 6D). APRIL, an inhibitor of adipogenic differentiation,^[^ ^34^ ^]^ was shown to be secreted from Pro‐MACs and received by committed preadipocytes, satellite cells, and B cells. Adropin, a fat‐burning hormone encoded by *ENHO*,^[^ ^35^ ^]^ was secreted from myocytes (fast) and interacted with pro‐NK/T cells through GPR‐19 in wild boars, indicating that adropin might play a direct regulatory role in the interaction between myocytes and immune cells.^[^ ^36^ ^]^ NPY stimulates the release of pro‐inflammatory mediators by mast cells in an autocrine manner. Collectively, we found more interactions of immune cells with other immune cells, committed preadipocytes (APRIL), and myocytes (ENHO) in wild boars than in domestic pigs.

To further explore the possible involvement of the cellular communication networks in the divergence of adipose phenotypes among pig breeds, we tested the roles of autocrine and paracrine mediation in FAP adipogenesis. We successfully showed that the autocrine signaling pathway, which was most intensive in Laiwu pigs (Figures S7F, S8A, Supporting Information), promoted adipogenesis but did not alter cell proliferation (Figure S8B, C, Supporting Information). In contrast, the IL‐1 autocrine signaling pathway, which was weakest in Laiwu pigs (Figures S7F, S8, Supporting Information), inhibited the proliferation and adipogenesis of FAPs (Figures S8B, C, Supporting Information), which supported our deduction that an autocrine effect may be involved in regulating intramuscular fat content. We also found that the expression levels of the IL‐1 receptor IL1RAP were higher in adipogenic precursors derived from Duroc pigs than in those from Laiwu pigs, under both growth and adipogenic differentiation conditions (Figure S8D, Supporting Information), whereas there was no significant difference in IL‐ secretion between the two breeds (Figure S8E, Supporting Information).

Regarding COL11A1^+^ tenocytes, interaction signaling pathways, including PDGF, periostin (POSTN), semaphorin 3 (SEMA3), and natriuretic peptide receptor 2 (NPR2), which have been positively correlated to fibrosis,^[^ ^37^ ^]^ were downregulated in Laiwu pigs relative to those in wild boars and Duroc pigs (Figure 6F). We also found that the osteocrin (OSTN) signaling pathway specifically originated from COL13A1^+^ tenocytes that were only observed in the neonatal skeletal muscle of Laiwu pigs (Figure 6G), and the addition of recombinant OSTN to adipogenic induction medium increased lipid production (Figure S8F, Supporting Information).

MACs function as a hub, secreting growth factors and cytokines for cell‐mediated immune responses, obesity‐related inflammation, and the proliferation and differentiation of satellite cells.^[^ ^38^ ^]^ Here, we analyzed various communication networks involving MACs in the three pig breeds (Figure S8G‐I, Supporting Information). In contrast with Laiwu pigs, Duroc, and wild boars specifically possessed ligand‐receptor signaling pathways that were secreted from MACs and used to regulated FAPs, such as PDGFRB, secreted phosphoprotein 1 (SPP1), and CALCR.^[^ ^39^ ^]^ Upon further exploration of the role of PDGFB in FAP adipogenesis, we found that the addition of recombinant PDGFB to culture medium promoted cell proliferation, but inhibited adipogenic differentiation, of FAPs in vitro (Figure S8H,I, Supporting Information). These results suggest that MACs might act to maintain the proliferative rather than the differentiated status of FAPs in neonatal skeletal muscle through the PDGFB‐PDGFRB ligand‐receptor interaction, thereby affecting adipogensis and subsequent fat accumulation in skeletal muscle. The Neu 2 subset showed high expression of type I interferon response genes, but did not secrete interferon I (Figure S8G, Supporting Information). Additionally, the ligand‐receptor interaction analysis revealed that Neu 2 exerted only a weak regulatory effect on FAPs through the resistin (RETEN) ligand‐receptor signaling pathway.

These results suggest that distinct resident‐cell profiles significantly affect ligand‐receptor interaction networks across cell types. In particular, the cell population of FAPs serving as a communication hub plays a critical role in the regulation of neonatal myogenesis, which may underpin the distinct muscle phenotypes.

### 2.6 Divergent Gene Expression Patterns in Skeletal Muscle–Derived Cell Types among the Pig Breeds

**Figure 7:**
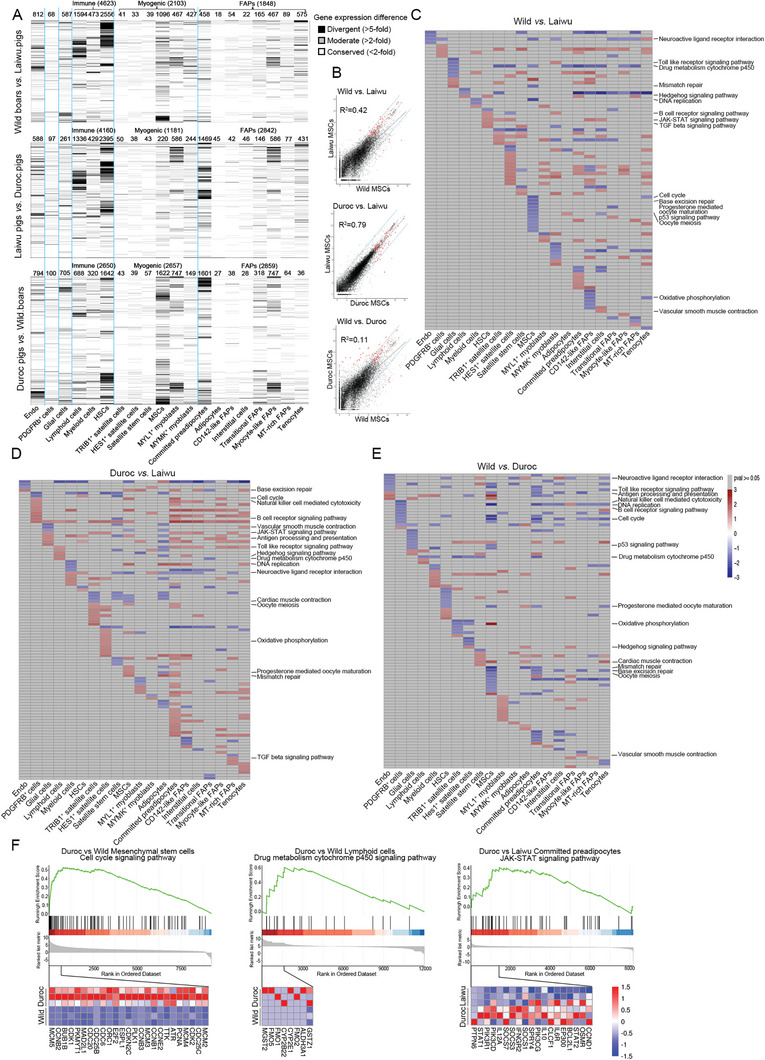
Divergent gene expression patterns across skeletal muscle–derived cell‐types among pig breeds. A) Patterns of expression change among pig breeds. Top, number of genes with expression divergence restricted to each cell types and broad class of cell types. B) Comparison of expression levels among pig breeds for MSCs. Genes outside the blue lines have highly divergent expression (>fivefold change) and include cluster‐ specific markers (orange dots). C‐E) The regulated pathways among pig breeds are displayed in a heatmap (C, wild boars versus Laiwu pigs; D, Duroc pigs versus Laiwu pigs; E, wild boars versus Duroc pigs). The color represents the NES for each pathway and gray indicates no significant difference in pathways. F) GSEA in cell cycle, drug metabolism cytochrome p450, and JAK‐STAT pathways. At the bottom of each panel shows a heatmap of representative genes found within the leading edge‐subset of the biological pathway. The color intensity represents median‐centered gene expression (FPKM) with red and blue representing highly and lowly expressed genes, respectively.

Identification of homologous cell types enables analysis of conservation and divergence of gene‐expression patterns across cell types. We found that homologous cell types comprising PDGFRb^+^ cells, TRIB1^+^ satellite cells, HES1^+^ satellite cells, satellite stem cells, committed preadipocytes, CD142‐like FAPs, interstitial cells, and MT‐rich FAPs showed high similarity in gene expression patterns among the pig breeds (**Figure** **7**A and Figure S9A,B, Supporting Information). In contrast, other homologous cell types, namely endothelial cells, glial cells, lymphoid cells, myeloid cells, HSCs, MSCs, MYMK^+^ myoblasts, MYL1^+^ myoblasts, adipocytes, transitional cells, myocyte‐like FAPs, and tenocytes exhibited large divergence in gene expression patterns (Figure 7A and Figure S9A,B, Supporting Information). HSCs possessed the largest number of genesshowing different expression (≥five‐fold changes) among the three breeds (Figure 7A). In particular, MSCs were the most divergent in terms of the similarity of gene expression patterns among breeds (*R* ^2^ = 0.42 between wild boars and Laiwu pigs; *R* ^2^ = 0.79 between Duroc pigs and Laiwu pigs; and *R* ^2^ = 0.11 between Duroc pigs and wild boars) (Figure 7B).

To identify key signaling pathways involved in skeletal muscle evolution, gene set enrichment analysis (GSEA) was employed to compare the gene expression patterns of each homologous cell subpopulation among the breeds (Figure 7C‐E and Table S2, Supporting Information). In contrast to wild boars, the MSCs of Duroc pigs retained their proliferative state, with upregulation of genes associated with proliferation‐related categories that guarantee DNA replication fidelity and proper cell proliferation, including the signaling pathways “base excision repair”, “DNA replication”, “mismatch repair”, “oocyte meiosis”, “cell cycle”, and “progesterone mediated oocyte maturation” (Figure 7E,F). These data will contribute to unraveling the molecular and cellular mechanisms of enlarged subpopulations of MSCs in Duroc pigs.

We also enriched signaling pathways implicated in the distinct muscle characteristics among the breeds, including categories related to the immune system, drug response, hypoxia response, and cardiovascular system, which was supported by a previous study demonstrating that these signaling pathways underwent selection during domestication.^[^ ^6^ ^]^ Furthermore, we showed that highly expressed genes in Duroc pigs were enriched in immune‐related categories at the single‐cell transcriptomic level, such as the B‐cell receptor signaling pathway and NK cell‐mediated cytotoxicity in PDGFRb^+^ cells, and the Toll‐like receptor signaling pathway in committed preadipocytes and tenocytes (Figure 7D,E). Moreover, Duroc pigs exhibited enhanced responses in drug‐related categories; specifically, enhanced drug metabolism by cytochrome p450 in lymphoid cells compared with wild boars and Laiwu pigs, and in TRIB1^+^ satellite, HES1^+^ satellite cells, and committed preadipocytes compared with wild boars (Figure 7E‐F). These differences could be due to the generations of exposure to high‐dose chemicals and/or drugs in Duroc pigs.^[^ ^6^ ^]^

In wild boars, a number of genes upregulated in the skeletal muscle were significantly enriched in the categories of hypoxia response and cardiovascular system. These included upregulation of cardiac muscle contraction genes in satellite stem cells, MSCs, adipocytes, and tenocytes compared with Duroc pigs, and upregulation of vascular smooth muscle contraction genes in myocyte‐like FAPs compared with Duroc pigs and in interstitial cells compared with Laiwu pigs (Figure 7C, E). These results demonstrated that skeletal muscle cell transcriptomes of wild boars possess characteristics that facilitated the adaptation of wild boars to harsh environments.

Relative to wild boars, neonatal MSCs from domestic pigs exhibited more activity in the p53 signaling pathway. The p53 signaling pathway controls stem cell self‐renewal and proliferation.^[^ ^40^ ^]^ We also found that neonatal MSCs from Duroc pigs exhibited significant repression in oxidative phosphorylation relative to wild boars (Figure 7E). Suppression of oxidative phosphorylation is necessary for self‐renewal in pluripotent stem cells.^[^ ^41^ ^]^ These data indicate that, in domestic pigs, larger numbers of MSCs retain active proliferation status compared with wild boars, which paralleled the results of pseudotime analysis.

**Figure 8:**
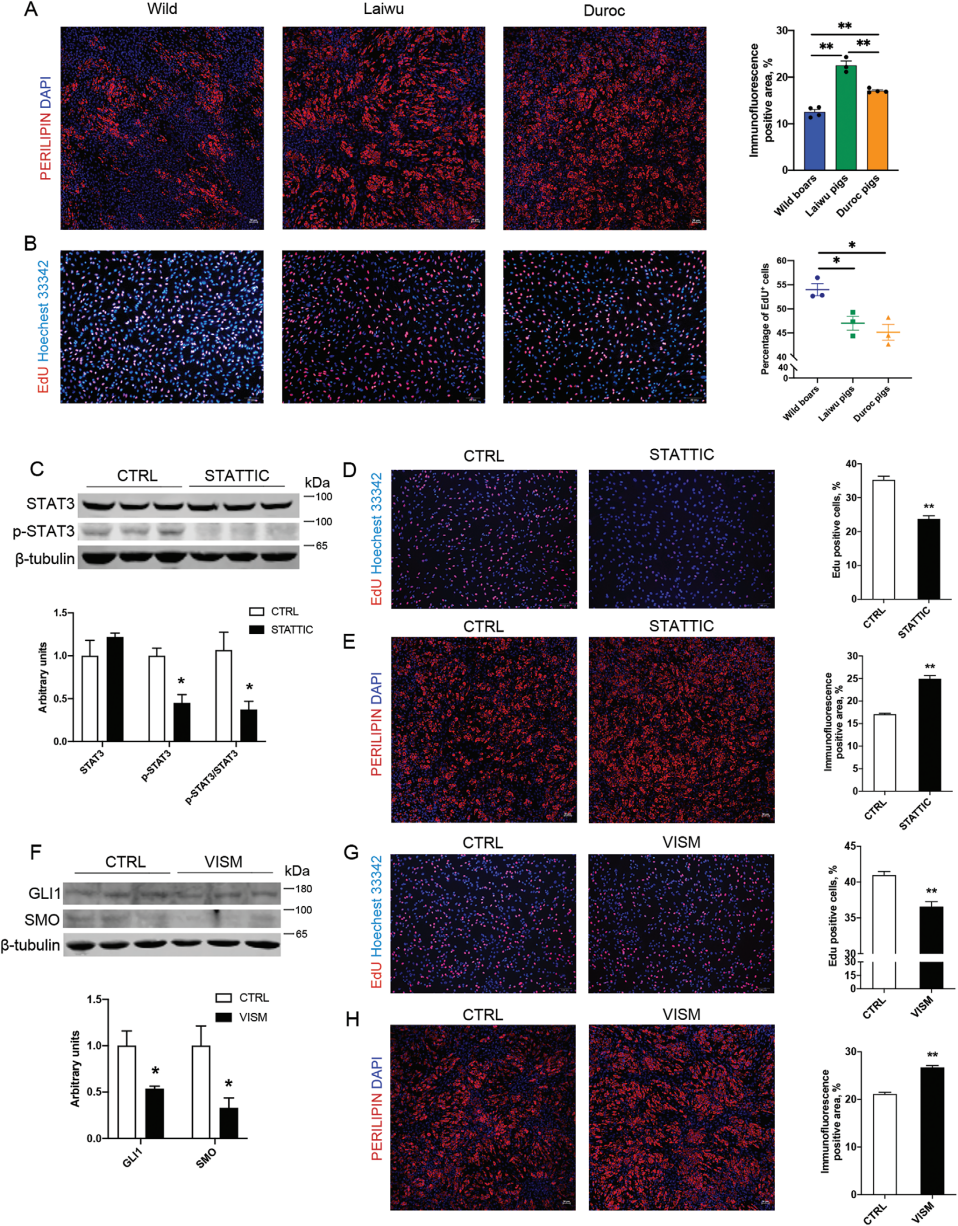
The role of JAK/STAT3 and Hedgehog signaling pathways in adipogenic differentiation. A) Immunofluorescent microscopy analysis of perilipin 1 in adipogenic precursors from wild boars, Laiwu, and Duroc pigs after 9 d of adipogenic differentiation. Scale bars, 50 µm. Immunofluorescence (perilipin 1) positive area was calculated at right. B) The proliferative potential of adipogenic precursors from wild boars, Laiwu, and Duroc pigs was measured by EdU staining. Scale bars, 50 µm. C) STAT3 and p‐STAT3 (Tyr705) levels of adipogenic precursors from Duroc pigs treated with DMSO control or 5 µM Stattic (a JAK/STAT signaling pathway inhibitor) in GM for 12 h. *n* = 3. D) Adipogenic precursors from Duroc pigs were treated with DMSO control or 5uM Stattic in GM for 12 h. Cell proliferation was measured by EdU staining. Scale bars, 100 µm. E) Immunofluorescent microscopy analysis of perilipin 1 in adipogenic precursors from Duroc pigs following 9 d of adipogenic induction and DMSO control or 5 µM Stattic treatment for the first 72 h. Scale bars, 50 µm. F) GLI1 and SMO levels of adipogenic precursors from Laiwu pigs treated with DMSO control or 10 µM Vismodegib (a Hedgehog signaling pathway inhibitor) in GM for 24 h. *n* = 3. G) Adipogenic precursors from Laiwu pigs were treated with DMSO control or 10 µM Vismodegib in GM for 24 h. Cell proliferation was measured by EdU staining. Scale bars, 100 µm. H) Immunofluorescent microscopy analysis of perilipin 1 in adipogenic precursors from Laiwu pigs following 9 d of adipogenic induction and DMSO control or 10 µM Vismodegib treatment for the first 72 h. Scale bars, 50 µm. Data are presented as means ± SEM. An unpaired Student's *t*test was used. CTRL, contral; VISM, Vismodegib. *n* = 3, *P < 0.05, **P < 0.01.

Several signaling pathways restrain adipogenesis, such as Hedgehog, TGF‐β, and JAK‐STAT.^[^ ^42^ ^]^ Consistently, compared with wild boars and Duroc pigs, Laiwu pigs showed downregulation of the TGF signaling pathway in MT‐rich FAPs, the JAK‐STAT signaling pathway in committed preadipocytes, CD142‐like FAPs, and interstitial cells. Laiwu pigs also showed downregulation of the Hedgehog signaling pathway in MT‐rich FAPs compared with wild boars, and in CD142‐like FAPs compared with Duroc pigs, in particular (Figure 7C,D,F). An ex vivo culture study was conducted to demonstrate the divergence in adipose properties of FAPs from the three pig breeds. Upon adipogenic induction, FAPs from Laiwu pigs showed the highest adipogenic potential of the three breeds (**Figure** **8**A), which was consistent with the results of IMF content staining by perilipin 1 (Figure 8A). Additionally, we showed that the proliferation of FAPs was similar between Laiwu and Duroc pigs using the 5‐ethynyl‐2′‐deoxyuridine (EdU) approach (Figure 8B). To test whether these signaling pathways participate in mediating FAP adipogenesis in pigs, FAPs undergoing proliferation or differentiation were treated with specific chemical inhibitors to depress the Hedgehog or JAK/STAT signaling pathways, respectively (Figure 8C–H, the in Figure 8C–E were from Duroc pigs, while 8F‐H were from Laiwu pigs). It was previously shown that inhibition of the Hedgehog and JAK/STAT3 signaling pathways hampers the proliferation of FAPs derived from the neonatal skeletal muscle of pigs while promoting adipogenic differentiation. These data could help to clarify the mechanism underlying the stronger adipogenic potential and resultant high IMF in Laiwu pigs.

Collectively, we compared the gene expression patterns of muscle‐resident cells, which led to the characterization of MSCs as the most divergent cells among the three breeds. GSEA demonstrated that the gene expression patterns of muscle‐resident cells conserved signs of past environmental challenges, such as wild boars arising from natural selection and generations of Duroc pigs subjected to intense chemical or medicinal exposure.

### 2.7 Comparison of Skeletal Muscle Expression Profiles across Species

**Figure 9:**
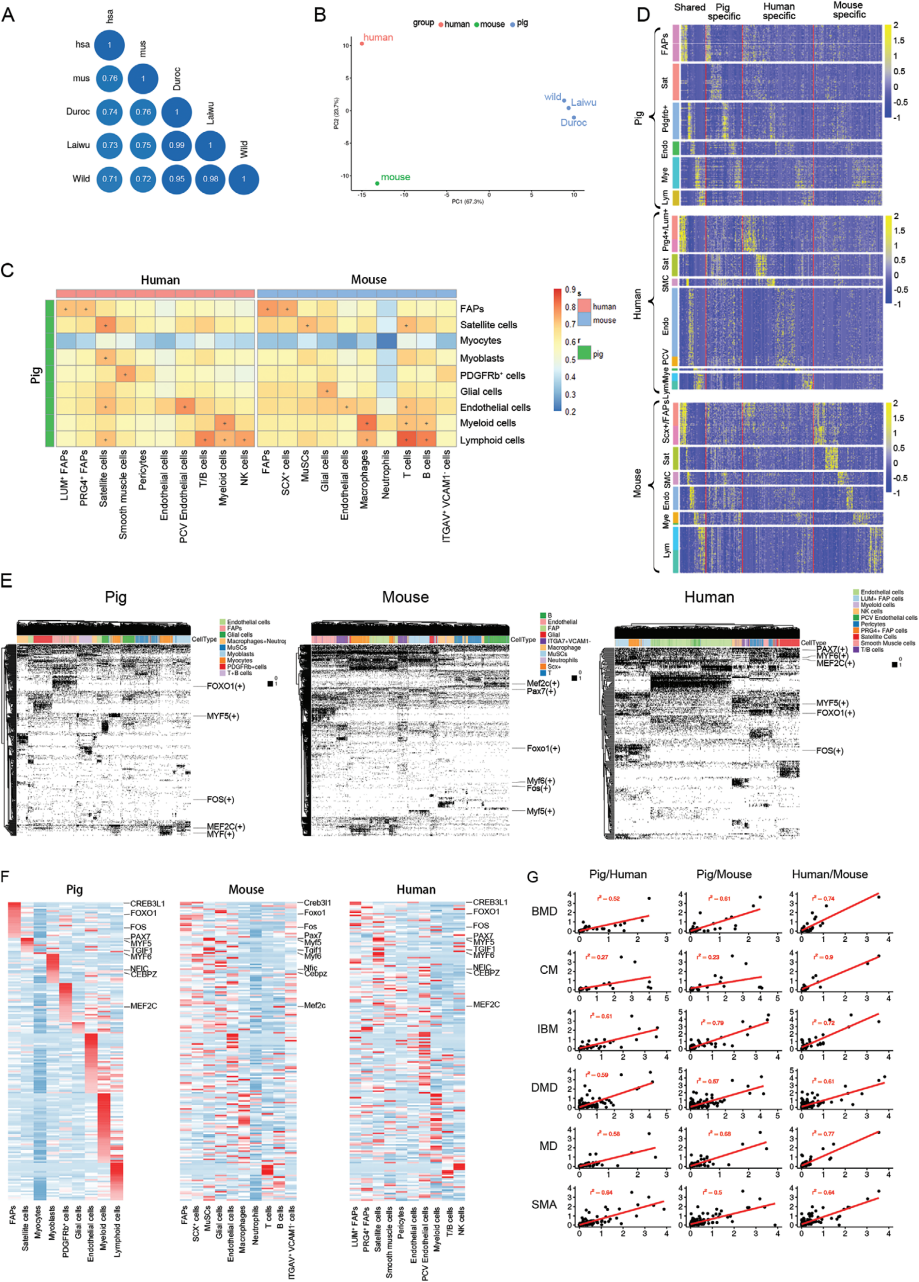
Comparison of skeletal muscle expression profiles across species. A) Correlation analysis of scRNA‐seq data of pig skeletal muscle (Duroc, Laiwu, and Wild) against human skeletal muscle,^[48b]^ as well as mouse skeletal muscle.^[9c]^ B) Principal component analysis to assess transcriptome similarity of pig skeletal muscle to human and mouse skeletal muscle. C) Comparison of cell types between human (column, left), mouse (column, right), and pig (row). Crosses indicate that the corresponding cell types are the best match. Color bars represent each cell type. D) Heatmap showing scaled expression levels of representative genes of pig skeletal muscle (top), human skeletal muscle (middle), or mouse skeletal muscle (bottom), with genes ordered into those common to species, or specific to either species for homologous cell types. E) Binary plots depicting active regulons in single cells from pig, mouse, and human datasets; regulons and cells are ordered by hierarchical clustering. F) Heatmaps of TF expression in pig, mouse, and the human respectively, identified in SCENIC analysis mentioned above. 186 regulons active in both species are shown. G) Pearson correlation of scRNA‐seq expression (UMI count) of susceptibility genes for (from top to bottom) Becker muscular dystrophy (BMD), Congenital myopathies (CM), Inclusion Body Myositis (IBM), Duchenne muscular dystrophy (DMD), Myotonic dystrophy (MD) and Spinal muscular atrophy (SMA) susceptibility genes. Species from left to right: pig versus human, pig versus mouse, and human versus mouse.

Owing to the unavailability of human and mouse data on the neonatal developmental stage, we were unable to make cross‐species comparisons at the same developmental stage. However, a cross‐species correlation analysis of whole muscle tissue indicated that similarities between our three pig breeds and humans/mice resembled those between mice and humans (*R* = 0.71–0.76; **Figure** **9**A, B). Skeletal muscle single‐cell transcriptomes of pigs were found to share high similarity with those of humans and mice, regardless of age (Figure 9C). Transcriptomes of almost all skeletal muscle cell types in pigs matched well with their homologues in humans and mice, suggesting that evolutionarily conserved transcriptomes might underlie the identities of muscle cell types across mammals. PDGFRb^+^ cells from pigs also shared high similarity with human smooth muscle cells. Interestingly, we also found that porcine endothelial cells were similar not only to their homologues in mice/humans but also to human satellite cells and mouse T cells. We further compared the transcriptional expression pattern of myogenic markers (*MYOD1*, *MYF5*, *DES*, *MET*, and *CDH15* in green text) and endothelial cell markers (*SELP*, *ITGAM*, *ITGB3*, *ACTA2*, *KDR*, and *CDH5* in red text) in porcine endothelial cells and human satellite cells (Figure S10, Supporting Information). We found that porcine endothelial cells and human satellite cells shared high similarity (*R* = 0.72, *P* < 2.2e‐16), mainly due to the similar expression patterns of *ITGAM*, *SELP*, *ACTA2*, *DES*, *CDH15*, and *MET*. However, in porcine endothelial cells, there was barely detectable expression of *PAX7* and *MYF5* and low‐level expression of *MYOD*, *MET*, *CDH15*, and *DES*. By comparison, *DES* was particularly highly expressed in endothelial cells from newborn wild boars. Our scRNA‐seq data implied a linkage between satellite cells and endothelial cells, which was also demonstrated in previous studies by Alfred Cossu's team.^[^ ^43^ ^]^

Next, we examined the differentially expressed genes in homologous cells among pigs, mice, and humans, and identified a total of 76 shared cell markers and 618 species‐specific cell markers (Figure 9D). Some canonical cell markers have general applicability across species. Here, we provide a foundational set of universal cell markers for the following cell types: *PAX7* and *CDH15* for satellite cells, *DCN* and *COL1A1* for FAPs, *PDGFRb* for PDGFRb^+^ cells, *CDH5* for endothelial cells, *CSF1R* for myeloid cells, and *CD3G* for lymphoid cells. We also characterized the following species‐specific markers: *LRP6* for porcine FAPs, *CALHM2* for human FAPs, *SLC48A1* for mouse FAPs, *F3* and *DPP4* for porcine muscle satellite cells, and *HEY1* for human muscle satellite cells. We anticipate that these may be useful for the identification and comparison of homologous cells among pigs, mice, and humans (Table S3, Supporting Information).

The concerted activity of co‐expressed TFs can be employed to define cell states.^[^ ^44^ ^]^ The functional activities of TFs in a cell may, in turn, be evaluated by examining the expression of their putative targets.^[^ ^45^ ^]^ In the present study, we analyzed TF activity to independently identify species‐specific and common TFs in skeletal muscle using pySCENIC.^[^ ^46^ ^]^ At the single‐cell transcriptome level, we observed that TF activity grouped independently and concordantly with a given cell type cluster. Overall, 376, 498, and 342 TFs were identified in skeletal muscle cells from humans, mice, and pigs, respectively (Figure 9E). Among these TFs, muscle‐resident cells across species shared a total of 186 active TFs. In addition to known key TF genes (e.g., *PAX7*, *MYF5*, *FOS*, *MEF2C*, *FOXO1*, *MYF6*, and *PKNOX1*), we uncovered gene regulatory networks that were not previously shown to be functional in muscle development (e.g., *CREB3L1*, *TGIF1*, *NFIC*, and *CEBPZ*) (Figure 9E). Moreover, we detailed the expression features of these TFs across cell types in pig, mouse, and human skeletal muscle, demonstrating that they were concordant with cell type‐specific TF activity (Figure 9F).

Finally, on the basis of the hypothesis that neonatal muscle‐resident cell profiles and transcriptomes determine adult muscle characteristics, we performed a cross‐species comparison of single‐cell transcriptome profiles of skeletal muscle to examine the conservation of susceptibility genes of the following human muscle diseases: Becker muscular dystrophy (BMD), inclusion body myositis (IBM), Duchenne muscular dystrophy (DMD), myotonic dystrophy (MD), congenital myopathies (CMs), and spinal muscular atrophy (SMA) ((Figure 9G). Transcriptional expression signatures of genes associated with BMD, IBM, DMD, MD, and SMA in human muscle showed high correlations with those of pigs and mice (*R* ^2^ = 0.52‐0.77). The respective correlation coefficients of pigs and mice with humans were similar in DMD (R^2^ human/mouse = 0.61, *R* ^2^ human/pig = 0.59) and SMA (*R* ^2^ human/mouse = *R* ^2^ human/pig = 0.64). In contrast, correlations of pigs and mice with human CM susceptibility genes differed (*R* ^2^ human/pig = 0.27, *R* ^2^ human/mouse = 0.90), indicating that mice are more suitable than pigs as a model for the study of CM disease. Collectively, we provided a foundational set of both cross‐species and species‐specific universal markers for muscle‐resident cell types. Pigs were found to share high similarity with humans in terms of single‐cell transcriptional expression profiles, especially for the human myopathies DMD and SMA.

## 3 Discussion

Skeletal muscle‐resident cell profiles change rapidly during embryonic and neonatal development. Although the cell profiles of human embryonic and adult muscle have been well studied,^[^ ^47^ ^]^ little is known about neonatal muscle‐resident cells. In this work, we compared data on three pig breeds to provide a panorama of neonatal skeletal muscle cell profiles. Through trajectory and ligand‐receptor interaction analyses, we showed that neonatal muscle‐resident cell profiles and the corresponding cell interactions vary among breeds. Our identification of new cell subpopulations and gene expression patterns that associate with divergent features of distinct muscle phenotypes, as well the cross‐species comparison, provide a resource for profiling muscle cell populations and targeting modulation of muscle development and regeneration.

One interesting finding was that neonatal skeletal muscle, in contrast to fetal and adult skeletal muscle across species,^[^ ^9^, ^24^, ^47^, ^48^ ^]^ displayed a more comprehensive repertoire of cell populations and subpopulations. In addition to MSCs, a panorama of myogenic lineages, including heterogeneous satellite cells, myoblasts, and myocytes, were found in neonatal skeletal muscle. Furthermore, we found that neonatal skeletal muscle satellite cells (PAX7^+^) not only include satellite stem cells but also encompass other two subpopulations with divergent myogenic potential (HES1^+^ and TRIB1^+^ satellite cells). Our study has demonstrated that the neonatal stage represents an excellent time point for revealing skeletal muscle‐resident cell profiles.

In contrast to FAPs in adipose tissue,^[^ ^9^, ^49^ ^]^ few studies have addressed the heterogeneity of FAPs in skeletal muscle.^[^ ^24^, ^50^ ^]^ Here, we discovered several FAP subpopulations in neonatal skeletal muscle, namely MT‐rich FAPs, myocyte‐like FAPs, COL13A1^+^ tenocytes, and proliferating preadipocytes. These subpopulations have distinct gene expression profiles, and their roles in myogenic development should be further investigated. In addition, two FAP subpopulations (DPP4^+^ and CXCL14^+^) previously identified in homeostatic muscle^[^ ^24^, ^50^ ^]^ were observed in our study, which we grouped as interstitial cells and committed preadipocytes, respectively.

In adult homeostatic skeletal muscle, cell turnover is limited.^[^ ^9c^ ^]^ In contrast, in neonatal skeletal muscle we found that, in addition to Pro‐MACs and pro‐NK/T cells, a number of stem‐like cells, including MSCs, interstitial cells (FAP subpopulation), and HSCs (immune subpopulation), as well as proliferating preadipocytes, were in active states of proliferation and differentiation.

In particular, we found a continuum between MSCs and FAPs characterized by shared canonical markers, such as CD73, CD90, and PDGFRA. In a previous study, the mesenchymal subtype of human fetal skeletal muscle (SkM.Mesen) cells expressed higher levels of mesenchymal/fibroblastic markers (e.g., PDGFRA and DCN) than main myogenic lineages, but lower levels than mesenchymal cells, while the mesenchymal/stromal population at the postnatal stage expressed the FAP marker PDGFRA.^[^ ^47^ ^]^ Considering these features of SkM.Mesen cells, namely reduced expression of myogenic markers and increased expression of mesenchymal cell markers (e.g., PDGFRA and DCN), we regard SkM.Mesen cells as MSCs.

It was previously shown that a small number of dual identity cells existed at a muscle‐tendon region in P0 neonatal mice, expressing both fibrogenic identity markers (*Pdgfrα, Dcn*) and myogenic markers (*Pax7, Myod1*), and these fibroblasts have switched on a myogenic program and fused into the developing muscle fibers along the muscle‐tendon junctions.^[^ ^51^ ^]^ Interestingly, we also detected *PDGFRa*/*PAX7* FAPs, accounting for ≈1% of total muscle‐resident FAPs, and also verified by co‐immunostaining in muscle sections. However, we do not have enough data to support the presence of this subpopulation yet, and it was s filtered out as suspected doublets by employing Scrublet.

We have also provided insights into the skeletal muscle‐resident cell profiles underpinning distinct skeletal muscle phenotypes. In contrast to domestic pigs, wild boars lack two subpopulations of FAPs (CYP1B1^+^ CD142‐like FAPs and CD9^+^ CD142‐like FAPs), but possess an extra transitional cell type (THY1^+^ satellite cells). Furthermore, we discovered breed‐specific cell types; for example, Duroc pigs possess proliferating preadipocytes and Laiwu pigs possess COL13A1^+^ tenocytes. Muscle tissue immune‐fluorescence staining showed that Duroc pigs have more proliferating preadipocytes in neonatal muscle than wild boars and Laiwu pigs, indicating that Duroc pigs might have the potential to develop higher IMF accumulation in future selective breeding. This possibility requires further investigation.

Pseudotime analysis of myogenic lineages shows that the pursuit of muscle growth rate and muscle mass has led to asynchronous myogenic differentiation. Myogenic lineages of neonatal Duroc pigs remained close to the original point of pseudotime trajectory. In particular, MSCs exhibited the most transcriptomic divergence across cell types among the breeds. Neonatal domestic pigs, especially Duroc pigs, had MSCs that were mainly in a proliferative phase, in contrast to wild boars, in which MSCs were mainly in a differentiation phase. Furthermore, MSCs of domestic pigs were involved in more ligand‐receptor interactions to control cell proliferation. Therefore, we can reasonably conclude that neonatal skeletal muscle MSCs of domestic pigs are distributed more closely to the original status in the trajectory compared with those of wild boars.

This study had limitations. First, we did not trace cell types across developmental stages, which might have detailed causal relationships between resident cell profiles and distinct adult muscle characteristics. Second, given the very low proportions of some cell subpopulations (≤2%) and the resulting difficulty in the required cell sorting, we did not directly examine their roles in ex vivo culture. Third, we did not profile muscle‐resident cell populations of adult pigs because it was considered a nonpriority objective.

## 4 Conclusions

The neonatal stage serves as a window into the panorama of muscle‐resident cells from which novel and breed‐specific cell subpopulations were identified and mapped on the pseudotime trajectory of muscle cell development. We demonstrated that artificial selection has caused significant changes in muscle‐resident cell profiles. FAPs exert key roles via ligand‐receptor interactions across cell types to form distinct muscle characteristics in pigs. Furthermore, we anticipate that this cross‐species comparison of single‐cell transcriptome data from humans, mice, and pigs will serve as a resource for the continued advancement of our knowledge of the conservation and divergence of mammalian muscle ontology.

## 5 Experimental Section

### Ethics

All animal procedures followed the animal care guidelines approved by the Animal Care and Use Committee of China Agricultural University (ID: SKLAB‐B‐2010‐003).

### Antibodies and Key Reagents

Information for all antibodies and key reagents can be found in Table S4, Supporting Information.

### Animals

Neonatal wild boars, purebred Laiwu (obese‐type), and Duroc (lean‐type) were employed in the study. Laiwu (obese‐type) and Duroc (lean‐type) piglets were purchased from the Laiwu Pig Original Breeding Farm (Laiwu, China) and Beijing Pig Breeding Center (Beijing, China), respectively. The wild boars used for this research were obtained from the Tiancheng Farm, Yangling, Shaanxi Province, China (wild boars breeding license available). Three male piglets in each breed within 3 days‐old were selected and the three piglets of the same breed were selected from different litters.

### Cell Isolation

Single‐cell suspensions from the *longissimus dorsi* muscle were prepared by mechanical and enzymatic dissociation as previously described^[^ ^52^ ^]^ with minor modifications in order to minimize the enzymatic dissociation time of individual cell from the skeletal muscle tissue. Briefly, after euthanasia, 1.5 g of muscle tissue was dissociated from the *longissimus dorsi* muscle over the last rib, manually minced and digested with protease (0.17%, Sigma‐Aldrich, Louis, MO, USA) for 45 min and collagenase‐type XI (0.15%, Sigma‐Aldrich) for 45 min at 37 °C in a thermostatic shaker (90 r min^−1^). The digestion was quenched by DMEM/F12 supplemented with 10% FBS, and the supernatant was filtered successively with 100 µm and 40 µm sterile strainers (BD Biosciences, San Jose, CA, USA). Cells were collected by centrifugation at 1000 ×*g* for 5 min and recovered in a growth medium.

For single‐cell sequencing, single‐cell suspensions were subjected to Debris Removal Solution (Miltenyi Biotec, Bergisch Gladbach, Germany), Dead Cell Removal Kit (Tissue‐Tek, VWR, Radnor, USA), and RBC lysing buffer (Sigma‐Aldrich) to exclude debris, dead cells, and red blood cells, as well as ambient RNA, respectively.

For cell culture studies, freshly isolated single‐cell suspensions were plated in growth medium in dishes coated with collagen I (Sigma‐Aldrich) for different times to sort Adi‐lineage cells (adhesion for 2 h) and the cell supernatant was transferred to fresh culture dishes coated with collagen I and 72 h adherent Myo‐lineage cells were harvested.

### Cell Culture

The adi‐lineage cells and myo‐lineage cells were cultured in growth medium containing DMEM/F12 (Hyclone, Logan, UT, USA), 10% FBS (Gibco‐BRL, Grand Island, New York, USA), glutamine (2 mM, Gibco‐BRL), and bFGF (5 ng ml^−1^, Peptech, Burlington, MA, USA). When adi‐lineage cells reached 90% confluency, culture media was switched by adipogenic differentiation medium containing DMEM/high glucose medium, 10% FBS, insulin (10 g ml^−1^), 1‐methyl‐3‐isobutylmethyl‐xanthine (0.5 mM), and dexamethasone (l M), for 3 days and DMEM/high glucose medium, 10% FBS and insulin (10 g ml^−1^) for another 5 days to induce adipogenic differentiation. For myogenic differentiation, myo‐lineage cells were differentiated at 90% confluency in DMEM/F12 medium containing 2% horse serum for 5 days. The medium was changed every 2 days.

For recombinant protein treatments, FGF2 (10 ng ml^−1^, PeproTech, Rocky Hill, NJ, USA), OSTN (0.5 µg ml^−1^, Novoprotein, Suzhou, China), IL1A (0.5 ng ml^−1^, PeproTech), PDGFB (10 ng ml^−1^, Abcome, Cambridge, MA, USA), or dexamethasone (50 µM, Sigma‐Aldrich) dissolved in growth medium or adipogenic differentiation medium was used.

For inhibition of JAK/STAT3 or Hedgehog signaling, cells were treated with stattic or vismodegib (10 µM, MCE, Shanghai, China) in growth medium for 12 h or adipogenic differentiation medium for 72 h.

### Plasmids and Transfection

CYP1B1 cDNA fragments were cloned into pcDNA3.1 vector. Plasmid transfection was performed using Lipofectamine 3000 Transfection Reagent (Invitrogen, Waltham, MA, USA) according to the manufacturer's instruction. After 24 h transfection, transfected myogenic precursors were switched into adipogenic differentiation medium.

### EdU Staining

Cell proliferation was measured using EdU cell proliferation assay kit (RiboBio Co., Guangzhou, China) according to manufacturer's instruction. To measure the proliferative capacity of Adi‐lineage and Myo‐lineage cells, the cells were added with Edu (10 µM final concentration) for 2 h at 70% confluency. As for recombinant protein treatments, including stattic, vismodegib, FGF2, IL1A, OSTN, and PDGFB, adi‐lineage cells were treated with corresponding reagents above for 12 or 24 h before EdU staining. The proliferating cells were stained with Appollo in red, cell nuclear was stained with Hoechst 33 342 in blue, and cell proliferation rate was determined as the proportion of red to blue.

### Western Blotting

Total cell protein extracts were obtained by RIPA with protease inhibitor cocktail and phosphatase inhibitor mixture (Huaxingbio Science, Beijing, China). Protein concentration was determined using a BCA protein assays kit. Protein extracts of 30 *µg* was electrophoresed in 8–10% SDS‐PAGE. Western blotting analysis was performed using a standard protocol. β‐tubulin was used as a loading control. Information regarding antibodies used are listed in Table S4, Supporting Information. The images were detected with Odyssey Clx (LI‐COR Biotechnology, Lincoln, NE, USA) and quantified using AlphaImager software (2200).

### ELISA

IL‐1α levels in cell culture supernatants were determined using the commercially available ELISA Kit (Nanjing Jiancheng Bioengineering Institute, Nanjing, China). Colorimetric solutions were quantified using a PowerWave 340 Microplate Spectrophotometer (BioTek, Winooski, VT, USA) and ELISACalc software.

### Immunofluorescence

Tissues were fixed in 4% PFA, dehydrated, paraffin‐embedded, and sectioned at 3–5 µm thickness. Dewaxed sections were first microwaved in Sodium Citrate buffer (pH 6.0) and then washed with PBS and permeabilized with 0.5% Triton X‐100 for 20 min. Tissue sections were blocked in blocking buffer (Beyotime, Shanghai, China) at room temperature for 1 h. Primary antibodies were applied for overnight at 4 °C and fluorophore‐conjugated secondary antibodies for 1 h at room temperature. Coverslips were placed over sections with mountant and DAPI. Tissue sections were visualized with Nikon T inverted (Nikon, Tokyo, Japan) and Olympus FluoView confocal (Olympus, Tokyo, Japan) microscopes. For quantitative estimation, the cross‐sectional area (CSA) was calculated in the laminin‐stained *longissimus dorsi* muscle sections using ImageJ software.

### Single‐Cell RNA Sequencing

The single‐cell suspension was washed twice with 1× PBS + 0.04% BSA. The cell concentration was determined using a hemocytometer and adjusted to obtain the target concentration for the 10X Chromium chip loading. Single‐cell suspensions were then loaded into the 10X Chromium Controller to generate single‐cell gel beads in emulsions (GEMs), and the Single Cell 3′ Reagent Kit v2 was applied according to the manufacturer's protocol. After the generation of GEMs, reverse transcription reactions were engaged to generate barcoded full‐length cDNA. Then cDNA was recovered, purified, and amplified to generate sufficient quantities for library preparation. Following library preparation and quantitation, the libraries were sequenced on the Illumina HiSeq xten platform.

### Processing of Single‐Cell RNA‐Seq Data and Quality Control

Cell Ranger version 3.1.0 was used to process the raw data and align the sequencing reads (fastq) to the *Sus scrofa* transcriptome and quantify the expression of transcripts in each cell. This pipeline resulted in a raw unique molecular identifier (UMI) count matrix for each sample, which records the number of UMIs for each gene associated with each cell barcode. All cells were removed that had either fewer than 200 expressed genes or over 10% UMIs derived from the mitochondrial genome. In order to eliminate potential doublets, single cells with over 6000 genes detected were also filtered out. Finally, 60 040 single cells remained, and they were applied in downstream analyses.

For each sample, UMI count matrices were normalized to total cellular read count and to mitochondrial read count using linear regression as implemented in Seurat's “RegressOut” function. To eliminate batch effects among three duplicate samples of the same breed of neonatal pigs (Laiwu, Duroc, and Wild), the authors corrected for the batch effect using the “FindIntegrationAnchors” function and “IntegrateData” function in Seurat V3.^[^ ^53^ ^]^ The “FindIntegrationAnchors” was used to identity anchors from different samples. Through these anchors, the “IntegrateData” was used to integrate the datasets together.

### Filtering Cell Doublets

To remove possible doublets, Scrublet was applied^[^ ^54^ ^]^ to predict suspected doublets and then estimated by expression patterns of cell type‐specific markers.

### Dimensional Reduction, Clustering and Annotation

“LogNormalize” and “vst” were applied to normalize and find variable features within the single‐cell gene expression data. It was implemented with “FindVariableFeatures” function in the Seurat package by setting the valid value of average expression as a range from 0.05 to 5 and that of dispersion as no less than 0.5. The “RunPCA” function in the Seurat package to perform the principle component analysis (PCA) on the single‐cell expression matrix with genes restricted to highly variable genes (HVGs) was then employed. Based on the PCElbowPlot, a certain number of principal components (PCs) for the clustering analysis when that number reached to the baseline of the standard deviation of PC was picked. We then cluster the cells using the “FindClusters” function in the Seurat package. Cell clusters were visualized using t‐SNE or UMAP plots. For sub‐clustering analysis, a similar procedure was applied including the variable genes identification, dimension reduction, cell integration with Harmony, and the clustering identification to the restricted cluster derived from the overall analysis. The cell populations were annotated based on the expression pattern of differentially expressed genes (DEGs) and the well‐known cellular markers from the literature. To identify the DEGs of a cluster relative to all other clusters, the “FindAllMarkers” function in Seurat was used. Parameters provided for this function were set as following: differential expression threshold of 0.25 log fold change using Wilcoxon rank sum test with *P* < 0.05 following Bonferroni correction.

### Comparison Dendrograms and Heatmaps Across Cell Types Among Pig Breeds

To compare similarities in cell types among the same breeds or between different breeds, average gene expression was obtained for each cluster. dist() function and hclust() function were used to calculate the distance and cluster the cell types respectively. And the cell‐specific expression patterns by the heat map was showed.

### Mapping FAPs Subpopulations to Known Populations

The similarity of FAP subpopulations with known cell populations using SciBet (v1.0),^[^ ^55^ ^]^ a tool for cell type identification to predict FAP subpopulations was compared. Thereby, the similarity score of each FAPs subsets with known cell types was obtained. The reference datasets^[^ ^9a,c^ ^]^ were analyzed using the Seurat pipeline. A higher score meant that the cell subpopulations were more similar to those previously known populations.

### Monocle Trajectory and RNA Velocity Analysis

To map the differentiation of the FAP subpopulations, pseudotime analysis was performed with Monocle (v2.14)^[^ ^56^ ^]^ to determine the dramatic transitional relationships among cell types and clusters. The Monocle function relative2abs was used to convert TPM measurement into mRNAs per cell, and then the CellData Set object was created with the parameter “expressionFamily = negbinomial”. The variable genes were selected using the Seurat R package. Then the cells differentiation trajectory was inferred after dimension reduction and cell ordering with the default parameters except method was specified = “DDRTree” in the reduceDimension function. The root state was set according to cell Seurat identified cell cluster label and “BEAM” function was used to calculate branch‐specific expressed genes. To plot branch‐specific expression heatmap, we used Monocle implemented “plot_genes_branched_ heatmap” function and genes with qval < 1e^−4^ were regarded as input genes. Genes were further divided into five sets according to k‐means. To investigate gene functions in each gene clusters, Metascape (http://metascape.org/gp/index. html#/main/step1) to perform gene ontology (GO) analysis was used.

For Figure 2G, the gene expression matrix of CD9^+^ CD142‐like FAPs, MYOC^+^ CD142‐like FAPs, CYP1B1^+^ CD142‐like FAPs, interstitial cells, adipocytes, COL11A1^+^ tenocytes and COL13A1^+^ tenocytes to identify differentiation potential of CD142‐like FAP subpopulations was extracted.

For Figure 5C, a joint trajectory was built to compare the myogenic trajectories of pigs across breeds by extracting the gene expression matrix of myogenic lineage cells, including MSCs, satellite stem cells, myoblast cells (combined MYL1^+^ myoblast and MYMK^+^ myoblast) and myocytes (combined myocytes (fast) and myocytes (slow)) from three pig breeds. The trajectory was inferred by the method described above.

RNA velocity was performed to investigate potential inter‐relationship of myogenic lineage cells in each pig breed. BAM file containing all the myogenic cells was used in this pipeline. All the parameters were set as default. The result was visualized into UMAP plot.

### Cell Cycle Analysis

The CellCycleScoring function in Seurat to score the cell cycle phases of each single cell was performed. This function calculated the cell cycle score based on previously published canonical marker genes.^[^ ^57^ ^]^ The single cells highly expressing G2/M‐ or S‐phase markers were scored as G2/M‐ or S‐phase cells, respectively, and the cells not expressing any of the two categories of genes were scored as G1 phase.

### Ligand‐receptor Cell Communication Model

CellChat (v0.0.1)^[^ ^58^ ^]^ in predicting ligand‐receptor interaction and cell–cell communication networks from single‐cell transcriptome data was employed. From the CellChat results, cell–cell communication in different pathways were obtained. For a given cluster, a ligand or receptor was considered expressed if 30% of cells had a UMI value of greater than 0. And the interaction across cell types in each species was shown in the heatmap.

### Quantification of Expression Divergence

The average expression for each pair of 20 homologous cell populations in wild boars, Laiwu, and Duroc pigs are calculated. Average expression values were log2‐transformed CPM. The fold difference in transcriptional expression of the homologous cells between two pig breeds was calculated for all genes and thresholds was set to identify large (>five‐fold), moderate (two to fivefold), and small (<two‐fold) differences, respectively. A heatmap was generated showing expression differences across cell types. For each of 20 homologous cell populations and three major classes of cell populations (immune cells, myogenic cells, and FAPs), the number of genes was quantified that had >five‐fold change in at least one cell type in that class. Scatter plots and correlations were calculated to compare the expression divergence of homologous cells between two pig breeds. Genes outside the blue lines have highly divergent expression (log2 fold change > 5) and breed‐specific marker genes were labeled in red.

### Gene Set Enrichment Analysis

The gene set enrichment analysis for concerned gene list by the tool R package ClusterProfiler (v3.14.3) was conducted.^[^ ^59^ ^]^ The BH method was employed for multiple test correction. GO terms with a *P* value lower than 0.05 were considered as significantly enriched (GO terms with a *P* value greater than 0.05 are shown in gray in Figure 7C–E).

### Correlation Analysis of Single‐Cell Expression Profiles

Correlations of scRNA‐seq data of pigs against human^[^ ^48b^ ^]^ and mouse^[^ ^9c^ ^]^ skeletal muscle scRNA‐seq data were analyzed. First, the single cell expression matrix (log normalized) of human and mouse muscle tissue was downloaded, and the average homologous gene expression matrix was calculated for each species, and then the Pearson's correlations between species and pig breeds were calculated using these matrices, and results were deemed significant if the correlation *P* value was less than 0.01.

It was also compared the gene expression pattern of human muscle disease (Becker Muscular Dystrophy (BMD), Congenital myopathy (CM), Inclusion Body Myositis (IBM), Muscular Dystrophy, Duchenne (DMD), Myotonic Dystrophy (MD), and Spinal Muscular Atrophy (SMA)) susceptibility genes from Disgenet dataset (https://www.disgenet.org/search) across species of human, mouse and pig.

### Ortholog Gene Selection

The homologous genes of human, mouse, and pig were obtained by getLDS() function in biomaRt package (v2.42.1),^[^ ^60^ ^]^ and genes with gene names was only retained. After that, these homologous genes (9614) for downstream analysis and comparison was used.

### Comparison of the Major Cell Types of Pig, Human, and Mouse Skeletal Muscle at Single‐cell Resolution

First single‐cell data from human and mouse muscle tissue described above was processed. Seurat pipeline was used for dimensionality reduction and cell clustering. Then marker genes identified in the paper was used to define cell types, and the Pearson's correlation coefficients of cell types among different species were calculated and the correlations were shown in a heatmap. Crosses indicate that the corresponding cell types are the best match.

Marker genes for each cell type in human, mouse, and pig muscle tissues, respectively, using “Findallmarkers” with default parameters in Seurat was also calculated. And the mouse genes were converted to human homologous genes. For the same cell type, “shared genes” meant that the genes were relatively highly expressed across three species and “species‐specific genes” meant that the genes were relatively highly expressed in one species.

### Transcription Factor‐Related Gene Regulation Sub‐Network Analysis

An analysis of the regulatory network and regulatory activity of human, mouse, and pig data sets using pySCENIC (v0.10.3) was performed.^[^ ^61^ ^]^ The ranking databases and the Motif‐to‐TF annotation database for human and mouse were downloaded from cisTarget databases (https://resources.aertslab.org/cistarget/). For pigs, the same database as for human was used. The count matrices were used as input to identify co‐expression modules by the GRNBoost2 algorithm. Following this, pySCENIC could find enriched motifs for a gene signature and optionally prune targets from this signature based on cisTarget databases. Using the aucell function, the functional activity score of the regulon is measured as the area under the recovery curve (AUC). Then, the binary regulon activity score was obtained using the binarize function. Finally, 376, 498, and 342 regulons in human, mouse, and pig was identified, respectively. Among them, there were 187 regulons were found to be overlapping between the two species from this analysis.

### Statistical Analyses

All statistical analyses were performed in “R” and “SAS” (version 9.2) software. Data represent means ± pooled SEMs. The significance of differences was determined using the unpaired Student's t‐test as indicated, and differences with *P* < 0.05 were considered as statistically significant. Detailed statistical methods in this paper can be found above.

## Conflict of Interest

The authors declare no conflict of interest.

## Author Contributions

D.X. and B.W. contributed equally to this work. J.Y. conceptualized the project, supervised the study, and conducted quality control on the data. J.Y. and D.X. wrote the manuscript. D.X., B.W., K. Q., Y.W., X.Z., N.J., E.Y., J.W., and C.L. collected the samples. D.X.K.Q., Y.W., and R.Y. performed the experiments. D.X., J.Y., and S.G. analyzed the data. D.X., J.Y., and X.Z. assembled the figures. J.Y. and D.X. contributed to the interpretation of the results. M.L., M.D., J.W., G.F., S.G., D.X., and J.Y. discussed results and commented on the manuscripts.

## Supporting information

Supporting Information

Supplemental Table 2

Supplemental Table 3

## Data Availability

The raw sequence data reported in this paper have been deposited in the Genome Sequence Archive^[^ ^62^ ^]^ (Genomics, Proteomics & Bioinformatics 2021) at National Genomics Data Center (Nucleic Acids Res 2022), China National Center for Bioinformation/Beijing Institute of Genomics, Chinese Academy of Sciences (GSA: CRA011788) that are publicly accessible at https://ngdc.cncb.ac.cn/gsa.
