# Supplementary material for: Single‐Cell RNA‐Sequencing Provides Insight into Skeletal Muscle Evolution during the Selection of Muscle Characteristics
Source: Adv Sci (Weinh). 2023 Oct 23;10(35):2305080. doi: 10.1002/advs.202305080 (PMC10724408; doi:10.1002/advs.202305080)
Supplement: Supplementary file 1 — Supporting Information [file ADVS-10-2305080-s002.pdf]

ADVANCED SCIENCE
Open Access

Supporting Information

for *Adv. Sci.*, DOI 10.1002/advs.202305080

Single-Cell RNA-Sequencing Provides Insight into Skeletal Muscle Evolution during the Selection of Muscle Characteristics

*Doudou Xu, Boyang Wan, Kai Qiu, Yubo Wang, Xin Zhang, Ning Jiao, Enfa Yan, Jiangwei Wu, Run Yu, Shuai Gao, Min Du, Chousheng Liu, Mingzhou Li, Guoping Fan and Jingdong Yin**

Supporting Information

Single-Cell RNA-Sequencing Provides Insight into Skeletal Muscle Evolution during the Selection of Muscle Characteristics

*Doudou Xu #, Boyang Wan #, Kai Qiu, Yubo Wang, Xin Zhang, Ning Jiao, Enfa Yan, Jiangwei Wu, Run Yu， Shuai Gao, Min Du, Chousheng Liu, Mingzhou Li, Guoping Fan, and Jingdong Yin **

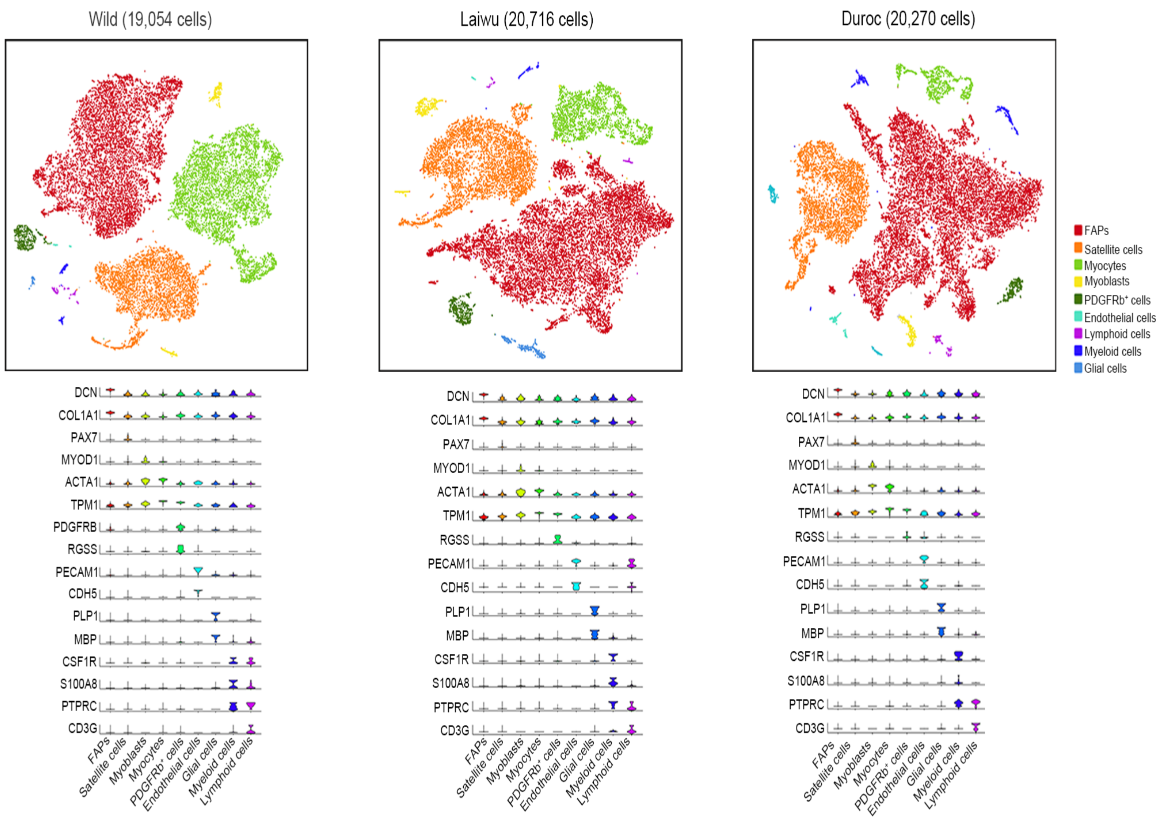

Figure S1. Related to Figure 1. Cell populations present in skeletal muscle tissues for each pig breed.

t-SNE plot revealed cellular heterogeneity with nine major populations and violin plots showed the expression levels and distribution of representative marker genes within each breed.

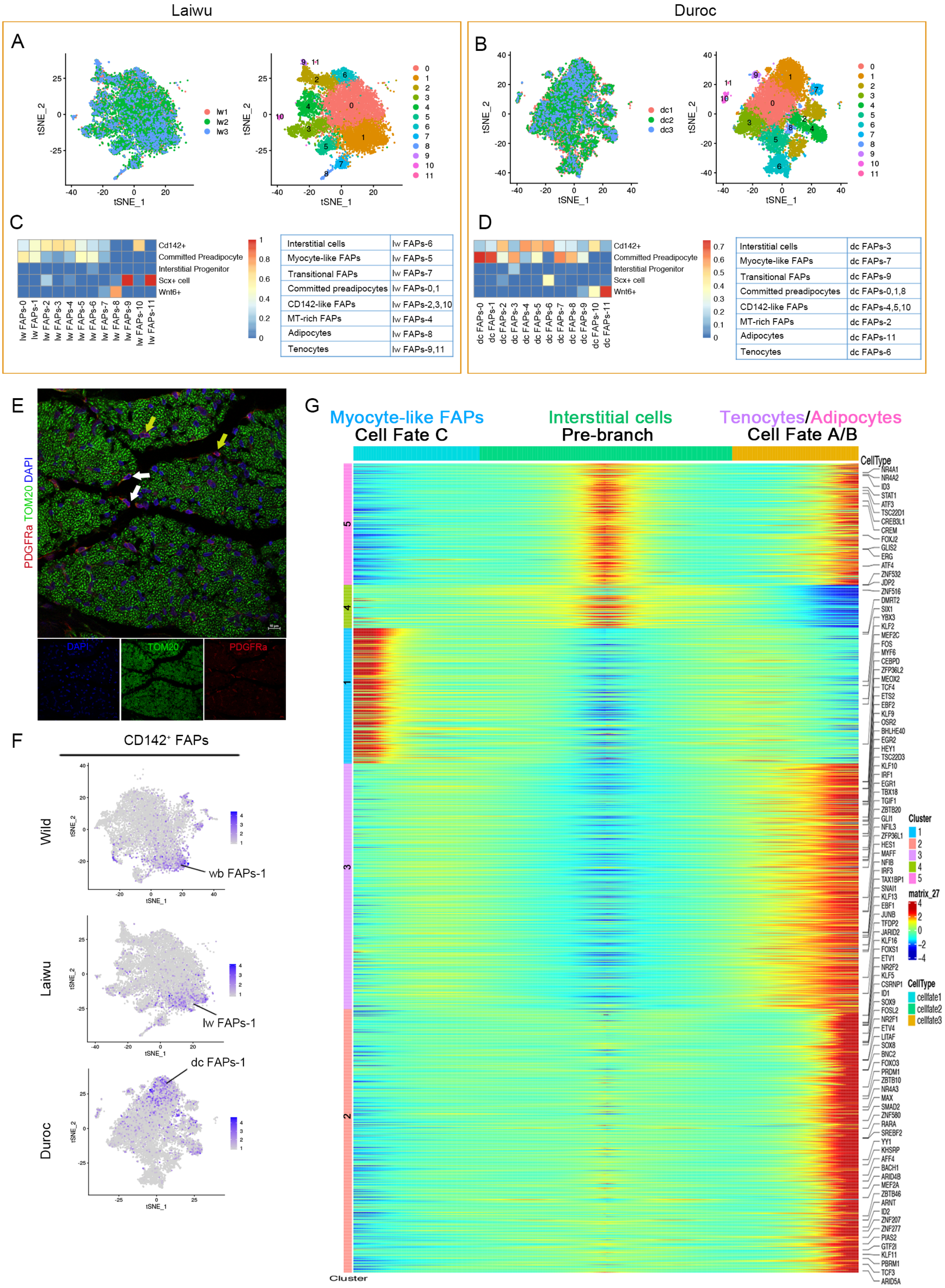

Figure S2. Related to Figure 2 and 3. Unbiased dissection of FAP subpopulations among pig breeds.

(A-B) FAPs from Laiwu pig (A) and Duroc pig (B) skeletal muscle were selected and re-analyzed.

(C-D) Comparison of observed Laiwu (C) and Duroc pig (D) muscle FAPs subsets to the FAPs reported in previous studies (Merrick et al., 2019; Schwalie et al., 2018; Giordani et al., 2019).
(E) Representative confocal images of skeletal muscle stained for FAP marker PDGFRa (red), DAPI (blue) and MT-rich FAPs marker TOM20 (Green). White arrowheads denote FAPs that are positive for the marker (scale, 10 μm). Yellow arrowheads mark FAPs that are negative for expression of the marker.
(F) Expressions of CD142 within wide boars, Laiwu pigs and Duroc pigs.
(G) Heatmap illustrating the TF of differentially expressed genes (DEGs) dynamics towards myocyte-like FAPs and tenocytes/adipocytes fate along pseudotime.

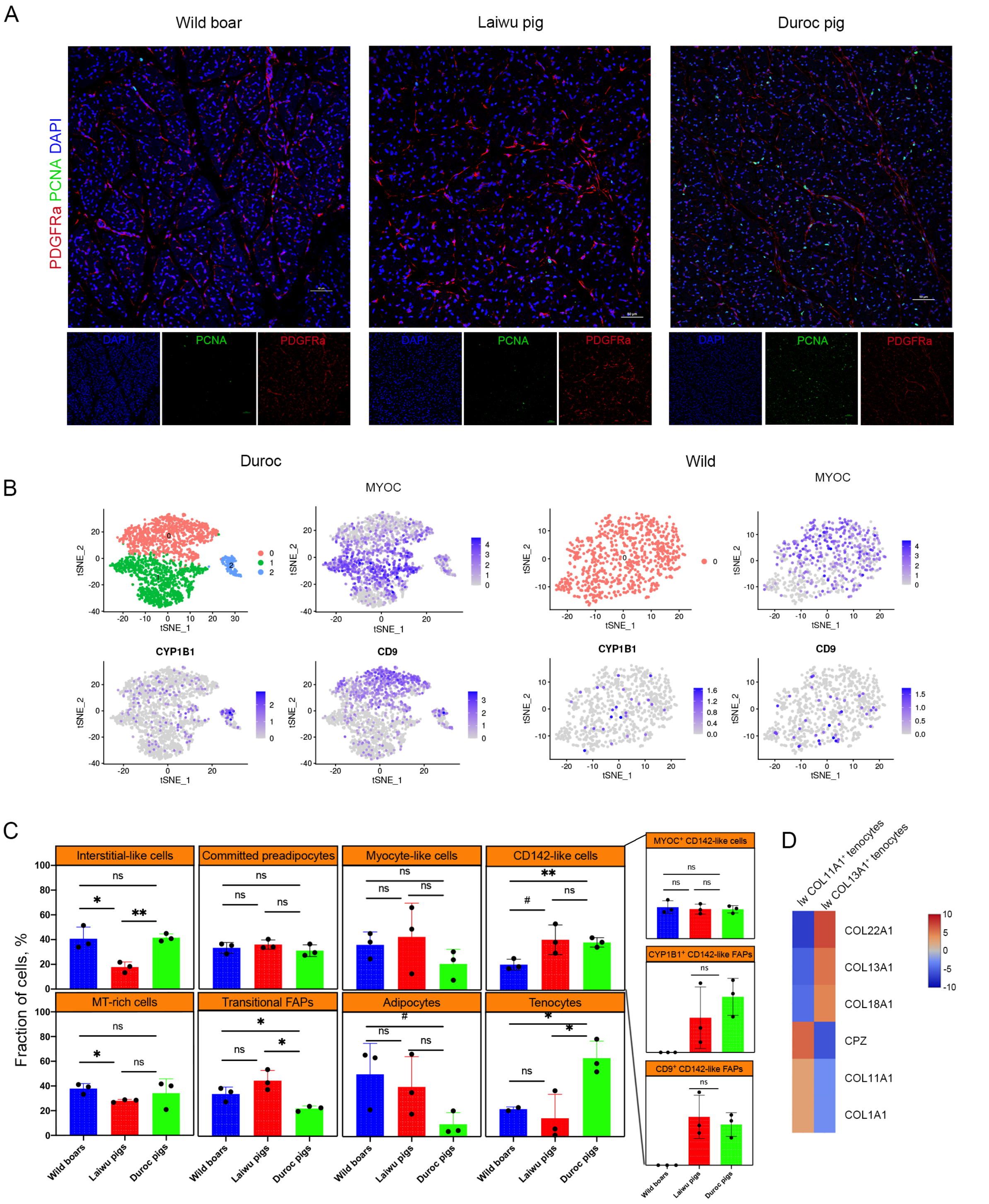

Figure S3. Related to Figure 2 and 3. Differences of FAP subpopulations among breeds

(A) Representative confocal images of PCNA (green) and PDGFRa (red)- immunostained skeletal muscle of wild boars, Duroc and Laiwu pigs (scale, 50 μm).

(B) CD142-like FAPs from Duroc pigs and wild boars were selected and re-analysed. t-SNE plot coloured by CD142-like FAPs subsets and showing the expression levels of markers for CD9^{+} CD142-like FAPs (*CD9*), CYP1B1^{+} CD142-like FAPs (*CYP1B1*) and MYOC^{+} CD142-like FAPs (*MYOC*). In wild boars, the CD142-like FAPs contain only MYOC^{+} CD142-like cells.

(C) Cell fractions of wild boars (blue), Laiwu pigs (red) and Duroc pigs (green) in each subpopulation. The average fraction (n = 3) and the SEM are shown on the bar plot. ns, not significant; Student's t test * $P < 0.05$, ** $P < 0.01$ and $^{\#}$ $0.05 < P < 0.10$.
(D) Heatmap showed differentially expressed genes between two tenocyte subpopulations (lw $COL11A1^{+}$ tenocytes and lw $COL13A1^{+}$ tenocytes).

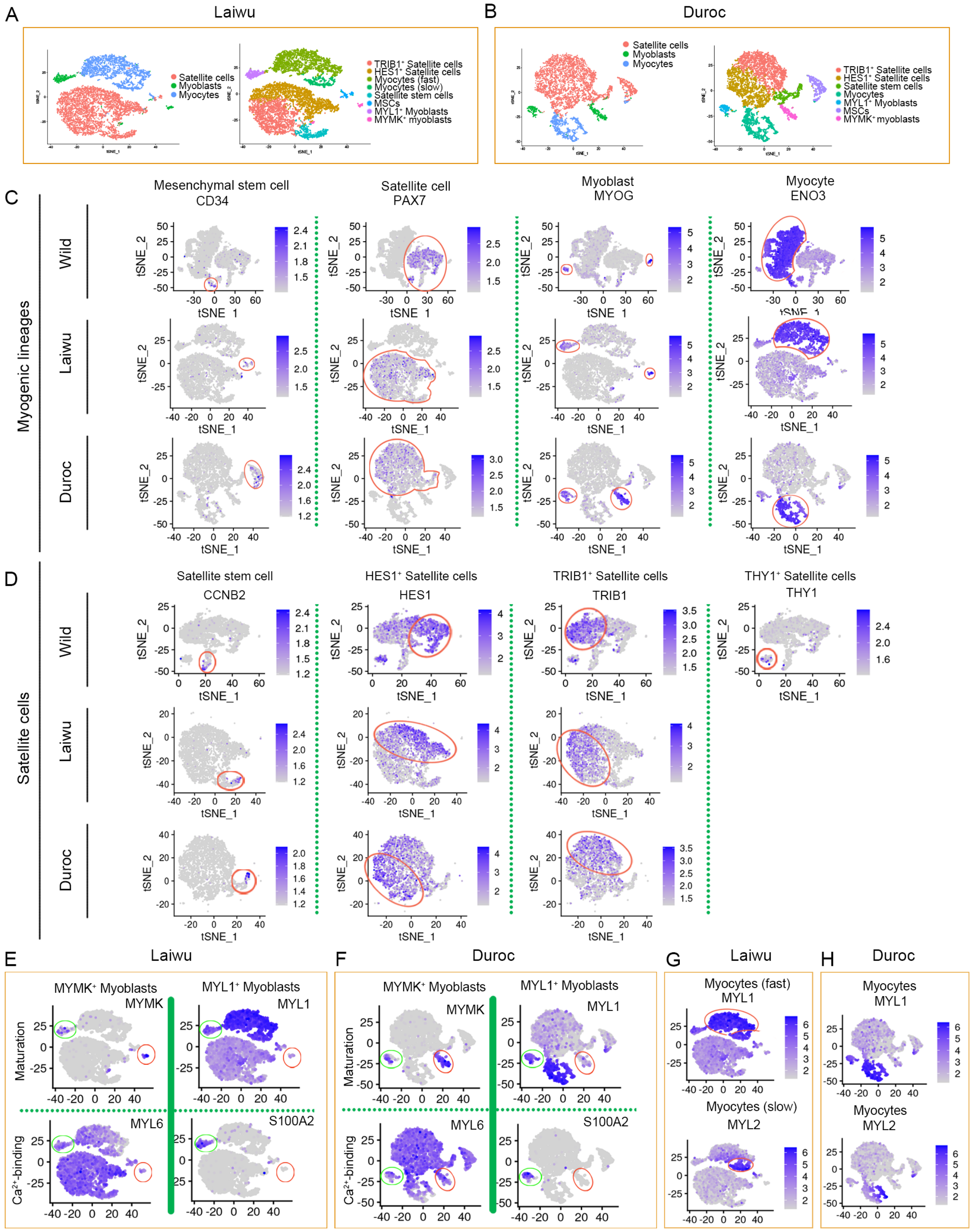

Figure S4. Related to Figure 4 and 5. Myogenic lineage subpopulations from wild boars, Laiwu and Duroc pigs

(A, B) Myogenic lineages (satellite cells, myoblasts and myocytes) from Laiwu (A) and Duroc pigs (B) were selected and re-analyzed. t-SNE plot colored by myogenic lineages (left) and manual classified cell subpopulations (right).

(C) t-SNE maps showing the expression levels and distribution of markers for mesenchymal stem cell (*CD34*), satellite cell (*PAX7*), myoblast (*MYOG*) and myocyte (*ENO3*) of wild boars (top), Laiwu pigs (middle), and Duroc pigs (bottom).
(D) t-SNE maps showing the distribution in the expression of selected markers of indicated satellite cell subpopulations of wild boars (top), Laiwu pigs (middle), and Duroc pigs (bottom).
(E-F) t-SNE maps showing the expression levels of genes related to muscle maturation and Ca^{2+}-binding capacity in two myoblast subpopulations of Laiwu (E) and Duroc pigs (F).
(G-H) Fast and slow myocytes recognized by myofiber type-specific markers (*MYL1* and *MYL2* respectively) in Laiwu (G) and Duroc pigs (H).

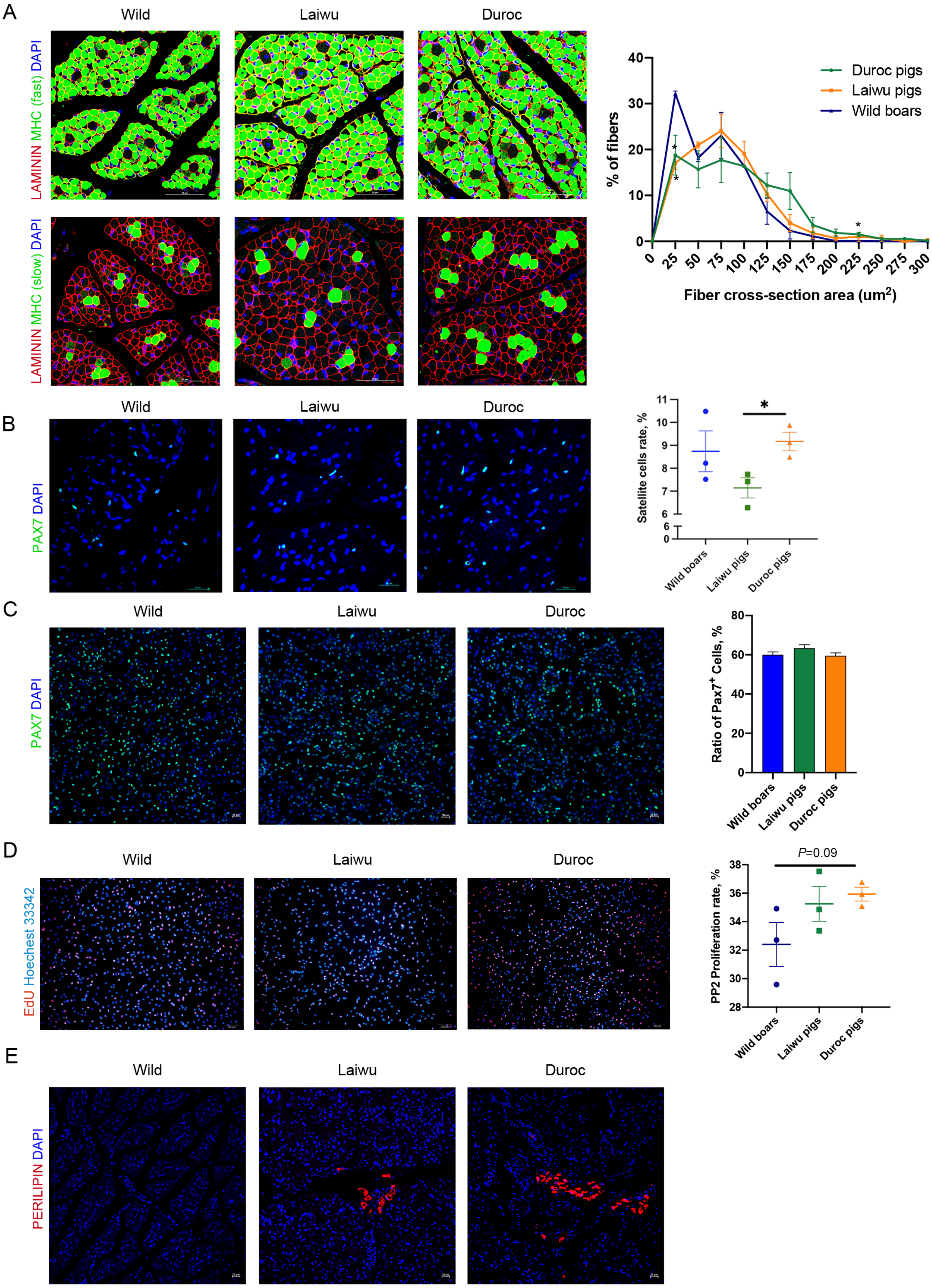

Figure S5. Related to Figure 4 and 5. The distribution of skeletal muscle fiber-type among pig breeds

(A) Representative confocal images of IHC staining for fast-twitch myofibers (green), slow-twitch myofibers (green), and Laminin (red). Scale, 50 μm. Neonatal skeletal myofiber size

(percentage) distributions of wild boars, Laiwu and Duroc pigs were measured by using ImageJ software. Only myofibers that contained centrally located nuclei were counted. $^{*}P < 0.05$.
(B) Representative confocal images of IHC staining for PAX7. Scale, 25 μm. n = 3, $^{*}P < 0.05$.
(C) Representative confocal images of myogenic precursors before myogenic induction after labeling PAX7 and DAPI. Ratio of $PAX7^{+}$ cells (normalized to DAPI cells) in wild boars (blue), Laiwu (red) and Duroc pigs (green). Scale, 50 μm. n = 3
(D) The proliferation capacity of myogenic precursors from wild boars, Laiwu and Duroc pigs were measured by EdU staining. Scale bars, 50 μm. n = 3
(E) Representative confocal images of transverse sections of longissimus dorsi muscle stained for perilipin from wild boars, Laiwu and Duroc pigs. Scale, 25 μm.

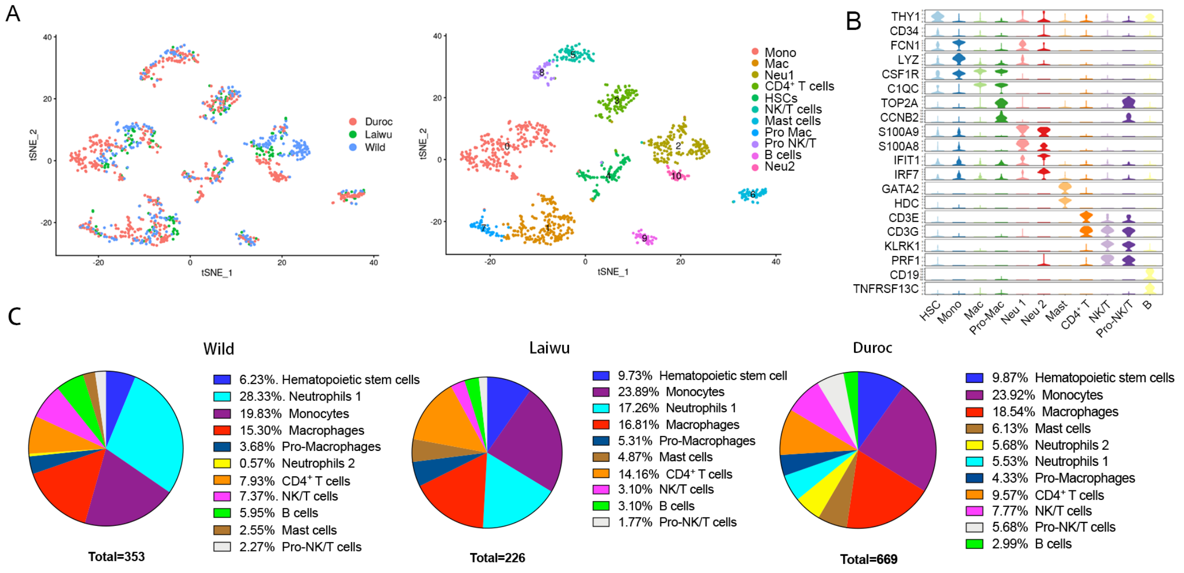

Figure S6. Immune cell subpopulations in the skeletal muscle

(A) Immune cells from skeletal muscle of wild boars, Laiwu pigs and Duroc pigs were selected and re-analyzed. t-SNE plot coloured by pig breeds (left) and immune cell subpopulations (right).

(B) Violin plots showing the expression levels and distribution of well-established cellular markers in cell populations identified as HSCs (*THY1* and *CD34*), monocytes (*FCN1* and *LYZ*), macrophage (*C1QC* and *CSF1R*), neutrophils (*S100A8* and *CSF3R*), mast cell (*HDC* and *GATA2*), B cell (*MS4A1* and *CD19*) and T cell (*CD3E* and *CD4*). HSCs: hematopoietic stem cells, Mono: monocytes, Mac: macrophages, Neu 1: Neutrophils 1, Pro Mac: proliferating macrophages, Pro NK/T: proliferating NK/T cells, Neu 2: neutrophils 2.

(C) Distribution of immune cell subpopulations in each pig breed.

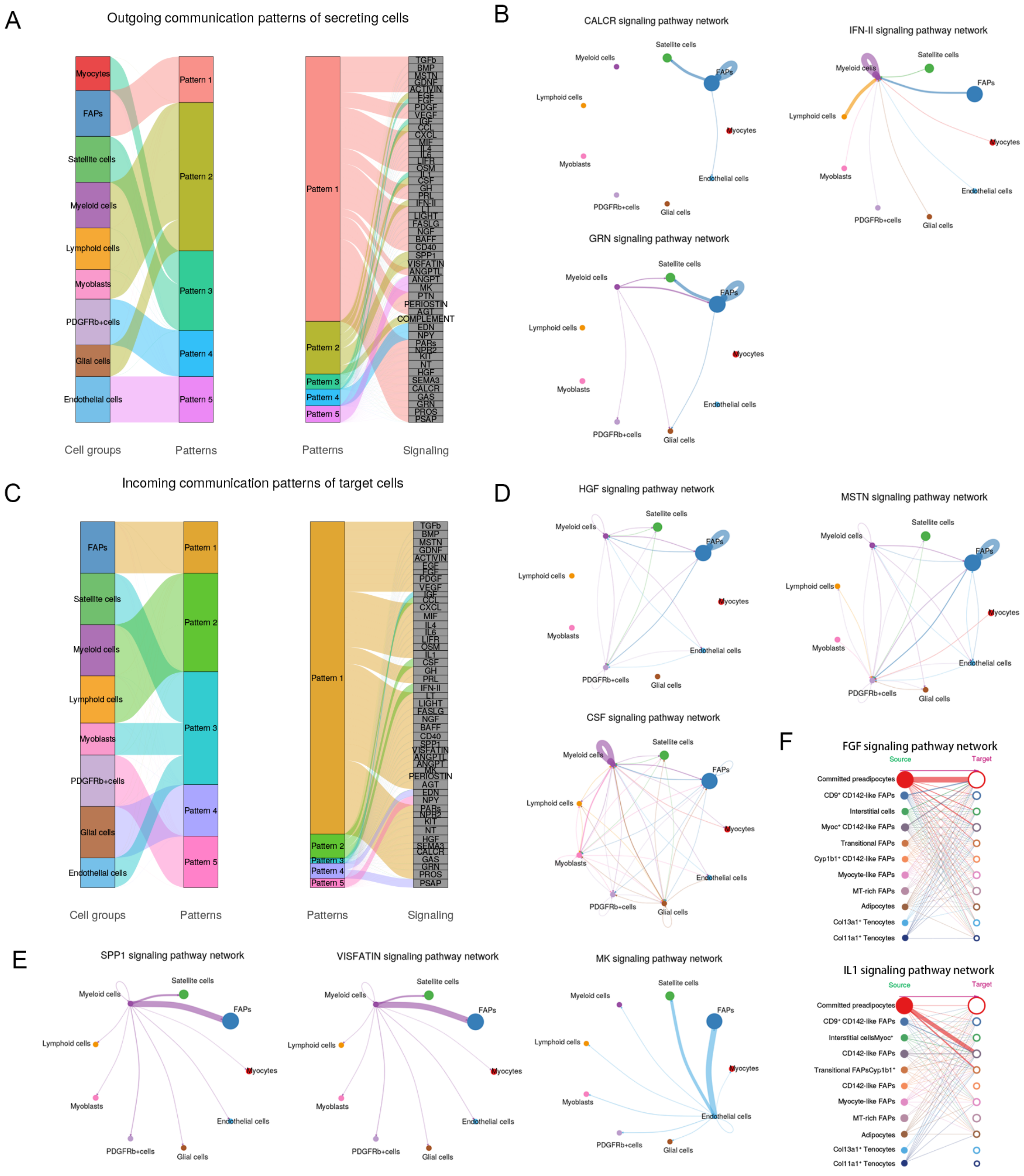

Figure S7. Related to Figure 6. CellChat analysis of the communications across skeletal cell type

(A) The alluvial plot showing outgoing communication patterns of secreting cells.

(B, D, E) Circle plot showing the number of statistically significant intercellular interactions for the selected pathway family of molecules. Each circle (color) represents one cell type; edges connecting circles represent significant intercellular signaling inferred between those cell types. Circles and edges are normalized to the number of cells for a given cell type and inferred strength of signaling, respectively.

(C) Incoming communication patterns of target cells.

(F) Hierarchical plot shows the inferred intercellular communication network for FGF (top) and IL1 (bottom) signaling within FAPs subsets of Laiwu pigs. Solid and open circles represent source and target, respectively. Circle sizes are proportional to the number of cells in each cell group. Edge colors are consistent with the signaling source.

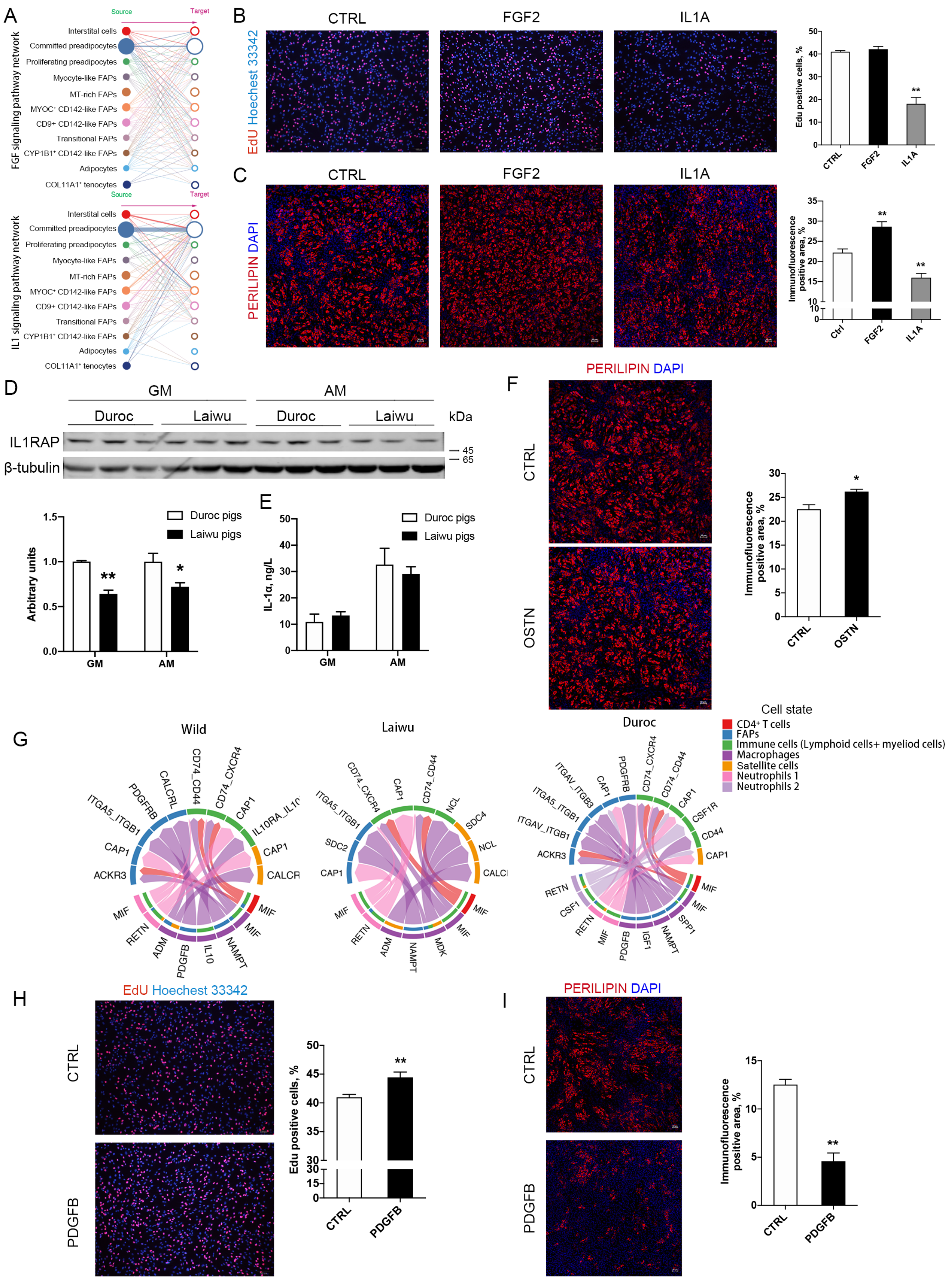

Figure S8. Related to Figure6. Differential intercellular signaling from immune cells among pig breeds

(A) Hierarchical plot shows the inferred intercellular communication network for FGF (top) and IL1 (bottom) signaling within FAPs subsets of Duroc pigs. Solid and open circles represent

source and target, respectively. Circle sizes are proportional to the number of cells in each cell group. Edge colors are consistent with the signaling source.

(B) Adipogenic precursors were treated with DMSO control, 10 ng ml^{-1} FGF2 or 0.5 ng ml^{-1} IL1A in GM for 24 h. Cell proliferation was measured by EdU staining. Scale bars, 100 μm. n = 3, $^{**}P < 0.01$.

(C) Immunofluorescent microscopy analysis of perilipin in adipogenic precursors following 9 d of adipogenic induction and DMSO control, 10 ng ml^{-1} FGF2 or 0.5 ng ml^{-1} IL1A treatment for the first 72 h. Scale bars, 50 μm. n = 3, $^{**}P < 0.01$.

(D) The expression of IL1RAP of adipogenic precursors from Duroc and Laiwu pigs were detected by western blotting in GM and AM. GM, growth medium; AM, adipogenic medium. n = 3, $^{*}P < 0.05$, $^{**}P < 0.01$.

(E) IL-1a levels in GM or AM cultured adipogenic precursors from Duroc and Laiwu pigs. n = 3.

(F) Immunofluorescent microscopy analysis of perilipin in adipogenic precursors following 9 d of adipogenic induction and DMSO control or 0.5 μg/ml OSTN treatment for the first 72 h. Scale bars, 50 μm. n = 3, $^{*}P < 0.05$.

(G) All possible ligand–receptor signaling pathways secreted from macrophages, neutrophils 1, neutrophils 2 and $CD4^{+}$ T cells, and received by satellite cells, FAPs, and immune cells.

(H) Adipogenic precursors were treated with DMSO control or 10 ng ml^{-1} PDGFB in GM for 24 h. Cell proliferation was measured by EdU staining. Scale bars, 100 μm. n = 3, $^{**}P < 0.01$.

(I) Immunofluorescent microscopy analysis of perilipin in adipogenic precursors following 9 d of adipogenic induction and DMSO control or 10 ng ml^{-1} PDGFB treatment for the first 72 h. Scale bars, 50 μm. n = 3, $^{**}P < 0.01$.

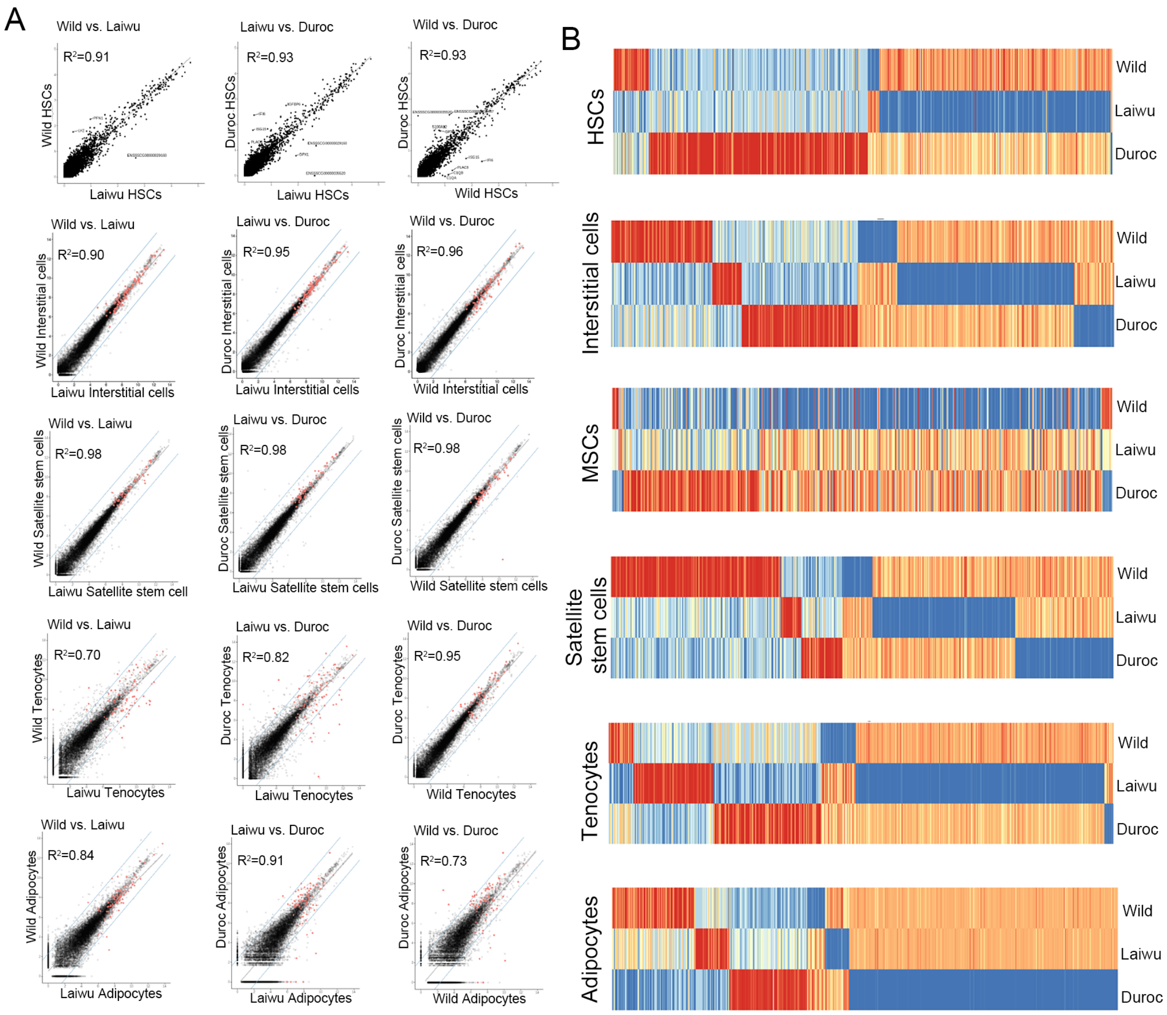

Figure S9. Related to Figure 7. The divergence and conservation of homologous cell types among pig breeds

(A) Comparison of expression levels among breeds for hematopoietic stem cells (HSCs), CD142-like FAPs, MSCs, satellite stem cells, tenocytes and adipocytes. Genes outside the blue lines have highly divergent expression (> 5 fold change) and include cell type-specific markers (orange dots).

(B) Relative expression in HSCs, CD142-like FAPs, MSCs, satellite stem cells, tenocytes and adipocytes in each pig breed (rows) of genes with preferential expression in the respective cell subpopulation, with genes ordered into those specific to pig breeds, or common to two pig breeds.

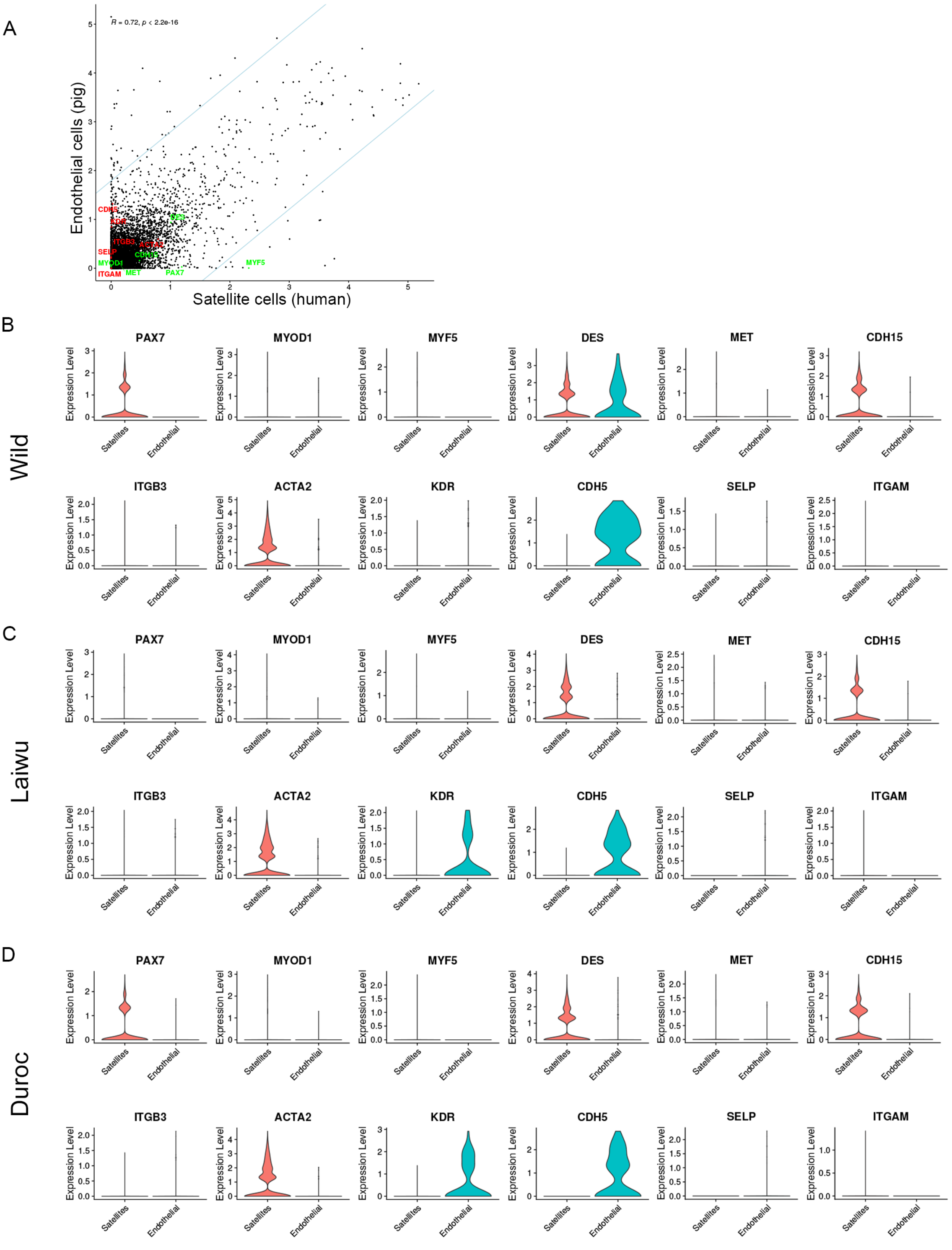

Figure S10. Related to Figure 9. Comparison of human satellite cells and porcine endothelial cells

(A) Comparison of expression levels between human satellite cells and porcine endothelial cells. Satellite cell markers were shown in green, while endothelial cell markers in red.

(B-D) Violin plots regarding the expression levels and distribution of representative markers in satellite cells and endothelial cells in wild boars (B), laiwu pigs (C) or Duroc pigs (D).

Table Captions for Table S1-4

Table S1: The quality information of sequencing data

Table S2: Enriched GSEA terms of biological processes on up-regulated genes of each homologous cell subpopulation among breeds （Upload seperately）

Table S3: Shared markers and species-specific markers of homologous cells among pigs, mice, and humans （Upload seperately）

Table S4: Reagent and resource used in this paper

Table S1. The quality information of sequencing data

Samples	Total sequencing reads	Median reads per cell	Total genes detected	Median UMI counts per cell
wb1	21,456,678	4,938	13,715	1,092
wb2	18,815,641	3,401	13,328	1,059
wb3	28,044,982	3,842	14,378	1,117
lw1	43,460,294	6,245	14,613	1,658
lw2	44,528,354	4,944	14,468	1,114
lw3	50,818,044	13,515	13,764	2,255
dc1	43,852,195	5,722	14,801	1,470
dc2	41,672,612	5,655	14,733	1,498
dc3	26,653,998	6,335	13,215	1,600

Table S4. Reagent and resource used in this paper

REGENT OR RESOURCE	SOURCE	IDENTIFIER
**Antibodies**		
Anti-PDGFRa	CST	Cat#: 3174
Anti-Pax7	DSHB	Cat#: Pax7
Anti-PCNA	Novus Biologicals	Cat#: NB500-106
Anti-Ki67	Abcome	Cat#: ab15580
Anti-Laminin	Sigma	Cat#: L9393
Anti-Cox 1	Abcome	Cat#: ab14705
Anti-Acta 1	Invitrogen	Cat#: MA5-11869
Anti-MHC (fast)	Leica	Cat#: NCL-MHCf
Anti-MHC (slow)	Leica	Cat#: NCL-MHCs
Anti-Mouse IgG-Alexa Fluor 488	Abcome	Cat#: ab150105
Antibody to Rabbit IgG -Alexa Fluor 647	Abcome	Cat#: ab150075
Antibody to Rabbit IgG -Alexa Fluor 594	Abcome	Cat#: ab150076
**Critical Commercial Assays**		
Chromium Single Cell 3' Library & Gel Bead Kit v2	10X Genomics	1000075
Chromium Chip B Single Cell Kit	10X Genomics	1000073
Chromium i7 Multiplex Kit	10X Genomics	120262
Debris Removel Solution	Miltenyi Biotec	130-109-398
Dead Cell Removal Kit	Miltenyi Biotec	130-090-101

Red Blood Cell Lysing Buffer Hybri-MaxTM	Sigma	Cat#: R7757
**Software and Algorithms**		
Cell Ranger (v3.1.0)	10X Genomics	https://github.com/10XGenomics/cellranger
Seurat (v3.0)	Stuart et al., 2019	https://github.com/satijalab/seurat
Monocle (v2.14)	Qiu et al., 2017	https://github.com/cole-trapnell-lab/ monocle-release
GSEA (v1.2)	Subramanian et al., 2005	https://www.gsea-msigdb.org/ gsea/index.jsp
ClusterProfiler (v3.14.3)	Yu et al., 2012	https://github.com/YuLab-SMU/clusterProfiler
SciBet (v1.0)	Li et al., 2020	https://github.com/PaulingLiu/scibet
CellChat (v0.0.1)	Jin et al., 2020	https://github.com/sqjin/CellChat
biomaRt (v2.42.1)	Durinck et al., 2009	https://github.com/grimbough/biomaRt
pySCENIC (v0.10.3)	Sande et al., 2020	https://github.com/aertslab/pySCENIC
Scrublet (v 0.2.1)	Wolock et al., 2019	https://github.com/swolock/scrublet
R (v3.6.1)		https://www.r-project.org/
Python (v3.7.1)		https://www.python.org/
